# Iron from Co-Encapsulation of Defatted Nannochloropsis Oceanica with Inulin Is Highly Bioavailable and Does Not Impact Wheat Flour Shelf Life or Sensorial Attributes

**DOI:** 10.3390/foods12030675

**Published:** 2023-02-03

**Authors:** Rohil S. Bhatnagar, Xin-Gen Lei, Dennis D. Miller, Olga I. Padilla-Zakour

**Affiliations:** 1 Department of Food Science, Cornell University, Ithaca, NY 14853, USA; 2 Tata-Cornell Institute for Agriculture and Nutrition, Cornell University, Ithaca, NY 14853, USA; 3 Department of Animal Science, Cornell University, Ithaca, NY 14853, USA

**Keywords:** bioavailability, encapsulation, fortification, iron, microalgae, roti, sensory study, shelf life, wheat flour

## Abstract

Defatted green microalgae *Nannochloropsis oceanica* (DGM) is a rich source of bioavailable iron. However, its use in foods results in unacceptable color and taste development. Therefore, the purpose of this study was to investigate strategies to enhance the use of DGM in foods. DGM and inulin were encapsulated (EC) in an oil-in-water emulsion using high-pressure homogenization. To confirm iron bioavailability, C57BL/6 mice were fed an iron-deficient diet (ID) for 2 weeks. The mice were then fed one of the four diets: ID, ID + DGM (DGM), ID + EC (EC50 or EC100) for 4 weeks. To test the stability of DGM as an iron fortificant at two different fortification rates of 17.5 mg Fe/kg (50%) or 35 mg Fe/kg (100%), whole (DGM50/DGM100), encapsulated (EC50/EC100) and color-masked (CM50/CM100) DGM were added to wheat flour (WF) at two different temperatures: 20 °C and 45 °C and were examined for 30 days. Acceptability studies were conducted to determine sensory differences between rotis (Indian flat bread) prepared from WF/EC50/CM50/EC100. The mice consuming EC50/EC100 diets showed comparable iron status to DGM-fed mice, suggesting that encapsulation did not negatively impact iron bioavailability. Addition of EC to wheat flour resulted in the lowest Fe^2+^ oxidation and color change amongst treatments, when stored for 30 days. There were no differences in the overall liking and product acceptance of rotis amongst treatments at both day 0 and day 21 samples. Our results suggest that EC50 can be effectively used as an iron fortificant in WF to deliver highly bioavailable iron without experiencing any stability or sensory defects, at least until 30 days of storage.

## 1. Introduction

Iron deficiency anemia (IDA) is the most common nutritional deficiency in the world affecting over 1.9 billion people [1]. One of the top strategies to improve dietary iron intake has been to fortify foods with iron [2]. However, the current iron fortificants with high relative bioavailability often cause lipid oxidation, off-color development and metallic aftertaste in foods [3]. In turn, these organoleptic issues reduce shelf life and product acceptance [4]. Hence, there is a need to identify effective iron sources and (or) methods to add iron to foods that may help overcome these technical challenges.

Microalgae contain rich amounts of proteins, micronutrients, antioxidants and ω-3 fatty acids [5]. Therefore, they can be a promising value-added ingredient in foods. However, there are very few commercial foods in the market that utilize microalgae, as their strong green color, fishy aroma and texture are hard to overcome [6]. In fact, even at minute inclusion rates, microalgae have been shown to negatively impact the sensory and textural outcomes of food products and their acceptance levels [7,8,9].

We have previously demonstrated the effectiveness of defatted microalgae *Nannochloropsis oceanica* (DGM) combined with inulin as an iron source [10,11]. There is limited literature available on the effects of *Nannochloropsis* use in foods [12,13]. In one of those studies, the incorporation of up to 30% *Nannochloropsis* to fresh pasta resulted in products with inferior cooking quality (swelling index, firmness, stickiness and chewiness) and lower consumer acceptance [12]. While the intrinsic iron content of the *Nannochloropsis* used in these studies was not reported, our DGM contains as much as 2700 mg Fe/kg. Such high iron content may enable the addition of minute amounts of DGM to foods as an iron source without appreciable sensory impact. These effects have not yet been investigated. 

India’s contribution towards the global anemia burden is estimated to be as high as 25% [1]. Due to its mass consumption in India, wheat flour (WF) is one of the most fortified food vehicles [14]. Fortifying WF with DGM may benefit millions of people that consume it on a regular basis. However, this can be challenging as the natural taste, color and aroma of roti needs to be maintained for continued consumer acceptance. In addition, fortified flours usually have a shelf life of 3 months in India, but can often spoil within 3–4 weeks due to insect infestation or oxidation [15]. Therefore, storage stability to assess physicochemical changes over time is an important consideration.

To enable the use of DGM as an iron fortificant in WF, it would be essential to conceal its color, taste, flavor and odor. Encapsulation may mask some of those sensory attributes, while preventing the undesirable interaction of microalgal iron with wheat phytate. However, encapsulation itself can affect bioavailability, as has been noted by others [16,17].

Therefore, the objectives of this investigation were: (1) to encapsulate DGM and inulin in emulsion for sensory-masking; (2) to test the iron bioavailability of encapsulated DGM against non-encapsulated DGM in diet-induced anemic mice; (3) conduct a shelf-stability study over 30 days by addition of treated DGM (encapsulated or color-masked by TiO_2_) to wheat flour at two addition rates, providing 50% and 100% of the iron recommended by Indian fortification standards, stored at two temperatures, 20 °C and 45 °C; and (4) assess the sensory effects on rotis prepared from wheat flour fortified with encapsulated DGM at day 0 and day 21 of storage.

## 2. Materials and Methods

### 2.1. Materials

Microalgae was obtained from Cellana (Kailua Kona, HI, USA). Whole wheat flour (100% extraction rate, Aashirvaad brand, Kolkata, West Bengal, IND) and jasmine rice bits (Asian Best Red Elephant brand, TH) were purchased from a local supermarket. Citric acid was sourced from Duda Energy LLC (Decatur, AL, USA), maltodextrin from Sigma Aldrich (St. Louis, MO, USA), non-GMO soybean oil from Healthy Harvest (Berthoud, CO, USA), TiO_2_ from Pure Organic (Solana Beach, CA, USA), sodium hexametaphosphate from Alfa Aesar (Haverhill, MA, USA), whey protein isolate from Agropur (BiPro, Saint Hubert, Longueuil, CA), soy lecithin from ADM (Chicago, IL, USA) and inulin (Raftiline HP, long chain, degree of polymerization = 10–60, average of 25) from Beneo-Orafti (Parsippany, NJ, USA).

### 2.2. Preparation of DGM and Inulin Loaded Microcapsules

Microcapsules were synthesized by encapsulating DGM and inulin in an oil-in-water (o/w) emulsion using conventional homogenization methods [18]. Different formulations were examined to prepare the microcapsules and the best formulation was selected. Briefly, the lipid phase containing soybean oil, soy lecithin (4.65% w/w) and DGM (23.26% w/w) was heated to 80 °C under constant rigorous stirring using an ULTRA-TURRAX^®^ homogenizer (IKA T-25, Wilmington, DE, USA) for 5 min. An o/w emulsion was prepared by dispersing this lipid phase under high-shear homogenization (Ross HSM-100 LSK, Hauppauge, NY, USA) at 8100 rpm for 3 min; 10,700 rpm for 4 min; and then 11,600 rpm for 3 min into an aqueous solution of inulin (6.90% w/w), whey protein isolate (WPI, 4.92% w/w), maltodextrin (4.72% w/w), sucrose (0.98% w/w), sodium hexametaphosphate (SHMP, 1.48% w/w), citric acid (0.98% w/w) and TiO_2_ (11.41% w/w) (1:2.5, lipid phase:aqueous phase). Fine emulsification was achieved by passing this coarse emulsion through a high-pressure homogenizer at 30,000 psi (Mini DeBEE, South Easton, MA, USA). The resulting emulsion was freeze-dried (Harvest right, North Salt Lake, UT, USA) for use in experiments. The freeze-dried cake was ground to a fine powder using a Robot Coupe R30 T (Ridgeland, MS, USA). The test sample was produced at the Cornell Food Venture Center Pilot Plant in Geneva, NY.

### 2.3. Particle Size Measurements and Morphology

The Mean Particle Size (MPS) was measured by light scattering using a Malvern Mastersizer 3000 particle size analyzer (Malvern, UK). The refractive index of the emulsion was used for particle size determination. Each sample was analyzed three times. Optical images of the coarse emulsion were obtained on a compound microscope (DMIL LED, Leica, Buffalo Grove, IL, USA) fitted with a mobile phone (iPhone SE, Apple, Cupertino, CA, USA). Scanning electron microscopy was conducted to characterize the surface morphology of the freeze-dried microcapsules. The freeze-dried samples were mounted on alumina stubs using double adhesive tape and gold sputtered for 30 sec. The samples were then observed using a JCM-6000 benchtop scanning electron microscope, software version 2.4 (JEOL Technics Ltd., Tokyo, Japan).

### 2.4. Particle Yield

The particle yield after freeze-drying was calculated according to Zhong et al. [19]:

$$\mathrm{Particle}\;\mathrm{yield}\;(\%)=\frac{\mathrm{mass}\;\mathrm{of}\;\mathrm{freeze}-\mathrm{dried}\;\mathrm{product}}{\mathrm{non}-\mathrm{solvent}\;\mathrm{mass}\;\mathrm{in}\;\mathrm{emulsion}}\times 100$$

### 2.5. Animals, Diets and Management

Three weeks old C57BL/6 mice purchased from Jackson Laboratories (Bar Harbor, ME, USA) and housed in the animal facility at Cornell University were fed an iron-deficient control diet (ID, 7.7 mg Fe/kg). After 2 weeks, the mice were allotted into four groups (n = 8/group; 4 males, 4 females) based on initial body weight, litter, gender and blood hemoglobin (Hb) concentrations. The mice were fed continuously either ID, or one of the experimental diets: ID + DGM (DGM diet, 31.6 mg Fe/kg), ID + encapsulated DGM (50% fortification level or EC50 diet, 17.3 mg Fe/kg; 100% fortification level or EC100 diet, 29.6 mg Fe/kg) for 4 weeks. Animals were housed in a controlled environment (12 h daylight cycle, lights off at 18:00) and provided with food and water ad libitum. The jasmine rice was ground to a fine powder using a coffee grinder (Krups F203, Solingen, Germany). The freeze-dried encapsulated products were incorporated in the rice-based mouse diets by Envigo (Indianapolis, IN, USA). Besides modifications to their iron content, the diets met the nutritional requirements of rodents [20]. The composition of different diets is provided in Table S1 (supplementary). The animal experiment was approved by the Institutional Animal Care and Use Committee at Cornell University (No. 2007-0008).

### 2.6. Biochemical Measurements

Animals were deprived of food for 8 h overnight before determinations of growth performance and blood collection. For initial Hb determination, blood was drawn via tail bleeding and Hb was measured using the cyanmethemoglobin assay method (Pointe Scientific, Canton, MI, USA). At the end of the study, the animals were euthanized in carbon dioxide-filled chambers. Blood was collected via heart puncture using heparinized syringes. Hb was measured using a Beckman-Coulter AcT Diff 2 coulter counter (Brea, CA, USA). Blood samples were centrifuged at 3000× *g* for 15 min at 4 °C. The resulting plasma was stored at −20 °C until testing. Liver and spleen were collected for downstream processing and stored at −80 °C until further analysis. Plasma levels of total iron binding capacity (TIBC) was determined using a Dimension Xpand Chemistry Analyzer (Block Scientific, Bellport, NY, USA). Plasma levels of ferritin was measured using a murine enzyme-linked immunosorbent assay kit (Abcam, Cambridge, MA, USA), according to manufacturer’s instructions. Non-heme iron contents of the liver and spleen tissue were determined as previously described [21], except that 2% v/v thioglycolic acid was used in place of hydroxylamine hydrochloride and the samples were incubated with the chromogen reagent for 30 min. The mice experiments were conducted at the Molecular Nutrition laboratory in the Department of Animal Science at Cornell University.

### 2.7. Fortification and Storage

In addition to encapsulated DGM (EC), a color-masked (CM; 45% DGM, 55% TiO_2_) formulation was also prepared by spraying DGM with TiO_2_ solution using a fluidized bed sprayer (Yamato DL-42, Santa Clara, CA, USA). Whole DGM acted as the positive control. Microalgae (DGM, EC and CM) was added to whole wheat flour (negative control, WF) at a fortification level of 17.5 mg of iron or 35 mg of iron/kg flour, meeting 50% or 100% of iron fortification requirements as set forth by the Food Safety and Standards Authority of India [22]. DGM was added at 0.61% and 1.22%, EC was added at 4.38% and 8.75% and CM was added at 1.60% and 3.21%, to the whole wheat flour to meet 50% and 100% of iron fortification levels respectively (Table 1). To ensure homogenous mixing, the flours were mixed for 5 min using a KitchenAid counter-top mixer (Pro600, Benton Harbor, MI, USA). The fortified flour was stored in clean, poly bags with aluminum layer (UV blocker, moisture barrier, air-tight) for 30 days and out of direct sunlight at ambient (20 °C) and abusive (45 °C) temperatures. As fortified flours can spoil within 3–4 weeks [15], storage stability of the fortified flours was determined at 0, 15 and 30 days [23,24,25]. The macronutrient composition of the fortified flours was determined according to standard AOAC methods (Table S2, Supplementary). The experimental design is provided in Figure S1, Supplementary. All food and emulsion-related experiments were carried out in the Food Processing laboratory in the Department of Food Science at Cornell University.

### 2.8. Iron Content

The total iron content of the freeze-dried microcapsules and of the fortified flour was determined using inductively coupled plasma atomic emission spectroscopy, as described elsewhere [26]. The amount of ferrous iron (Fe^2+^) in the fortified flour was measured in triplicate using bathophenanthroline assay, as previously described [27].

### 2.9. Oxidative Stability

The formation of secondary lipid peroxidation products in wheat flour was determined in duplicate using the method of Buege and Aust [28]. Malondialdehyde (MDA) concentration was calculated using the molar extinction coefficient of 1.56 × 10^5^ M^−1^ cm^−1^ and results expressed as micromole MDA per gram of fat.

### 2.10. Colorimetry

Color analysis of control and fortified wheat flours was assessed in triplicate using a Konica Minolta Chroma Meter CR-400/410 (Optics Inc., Osaka, Japan). CIE (Commission Internationale de l’Eclairage) L*a*b* color system was used where L* represents luminosity (or lightness) and a* and b* represent chromaticity layers red-green and blue-yellow axes, respectively [29].

### 2.11. Moisture Content

Moisture content was determined according to the oven-drying method [30]. Samples were analyzed in duplicate and the results expressed as % moisture.

### 2.12. Sensory Acceptance Study

A sensory study was conducted during the coronavirus pandemic with a blind and untrained sensory panel, recruited from an existing pool of panelists with varying levels of familiarity with the test product and with no gluten allergies. The panelist demographics are provided in Supplementary Table S3. The panelists were provided with study instructions and samples of Indian unleavened flat bread (roti) made from four flour samples: WF, WF + 17.5 mg iron provided by EC (EC50), WF + 17.5 mg iron provided by CM (CM50) and WF + 35 mg iron provided by EC (EC100) via contactless delivery to their homes. Spring water and saltines were also provided as palate cleansers. The panelists were asked to electronically record their consent and responses using the RedJade software (version 4.0, RedJade Sensory Solutions LLC, Martinez, CA, USA) by the next day on overall acceptability, texture, flavor and other pertaining attributes using a 9-point hedonic scale, a 7-point just about right (JAR) scale, purchase intent (pre- and post-claim) and short open-ended questions [31]. The acceptability tests were performed on rotis prepared from Day 0 and Day 21 flour samples stored at room temperature (18–22 °C) to compare any detectable sensory differences in the flours upon storage. There were 59 panelists for the day 0 study and 53 panelists for the day 21 study. Sensory experiments were conducted according to protocols approved by the Institutional Review Board for Human Participants at Cornell University.

### 2.13. Rotis Preparation

Rotis were prepared by mixing 1000 g of WF and water (~590 mL) using a KitchenAid counter-top mixer (Pro600, Benton Harbor, MI, USA) at low speed for 2 min and then on moderate speed for another 2 min. For the test samples, appropriate amounts of material supplementing 17.5 mg or 35 mg iron were also added (Table 1). The dough was rested briefly. Then, 30 g of dough was rolled to a circular shape of approximately 2 mm thickness. The rolled dough was baked on a pre-heated griddle for 45 sec on either side and then puffed directly on the gas flame. The rotis were cooled and individually packed in airtight polypropylene bags (Ziploc, SC Johnson & Son, Inc., Racine, WI, USA) for sensory analysis.

### 2.14. Statistical Analyses

GraphPad Prism v 7.0 (La Jolla, CA, USA) was used for statistical analysis. The main effects of the diets on animals were analyzed using a *t*-test (unpaired, Welch correction). Analysis of variance (ANOVA) corrected for multiple comparisons using post-hoc Tukey’s test was used to compare differences between treatments in the shelf-life study. For sensory studies, ANOVAs corrected for multiple comparisons using the Brown-Forsythe and Welch correction tests were used. The normality of the hedonic distribution was determined using the Shapiro-Wilk test. To assess the uniformity in the variances, Spearman’s heteroscedasticity test was used. Penalty analysis was performed on JAR profiles using XLSTAT statistical software (Addinsoft, NY, USA) and threshold was set at 20% of the participant size. *p*-values less than 0.05 was considered statistically significant. Values are expressed as means ± S.E., unless specified.

## 3. Results and Discussion

### 3.1. Physical Stability, Morphology, Particle Yield and Encapsulation Efficiency of the Emulsion and Freeze-Dried Powder Encapsulates

**Figure 1:**
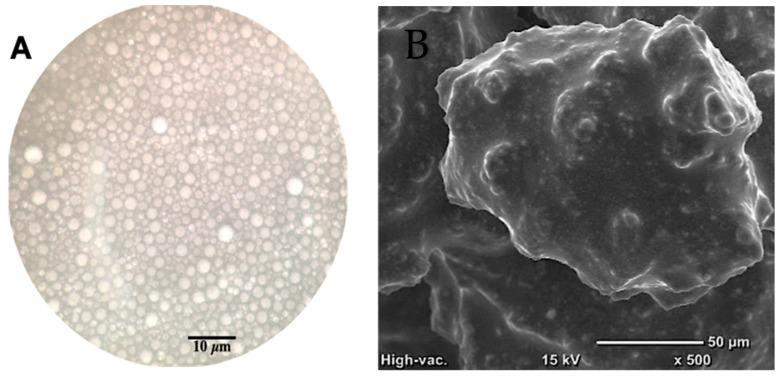
(A) Optical microscopy of the high-sheared emulsion (Scale bar: 10 μm); (B) SEM image of the freeze-dried encapsulated powder containing DGM.

Different formulations were examined to prepare the microcapsules. The formulations were rejected if they: (1) did not mask the sensory attributes of the DGM sufficiently and/or (2) yielded low DGM encapsulation (as measured through total iron content). Using the methodology of [19], the freeze-drying process yield for the best formulation was calculated to be 77.8%. The visualization of the emulsion under the microscope revealed that the o/w emulsion droplets were spherical in shape and tightly packed together (Figure 1A). Under the SEM, the electron micrographs of the freeze-dried microcapsules showed a non-porous surface morphology. Freeze-drying resulted in aggregated particles, as the particle size seemed larger than o/w emulsion droplets (Figure 1B). The mean diameter over volume, D(4,3), of the o/w DGM-inulin emulsion droplets was determined on days 0, 21 and 42 (Table 2). The day 0 D(4,3) value of the emulsion was 0.63 μm and it did not appreciably change after storage for 42 days at 4 °C. This suggests that the emulsion was stable against Ostwald ripening and droplet coalescence. While this emulsion had a total lecithin concentration of 1.4%, these results agree with previous findings that a soybean o/w emulsion can be stabilized by using lecithin at a concentration greater than 1.2% (w/w) [32]. In addition, conjugates of whey protein isolate and maltodextrin have been shown to stabilize microcapsules against aggregation over longer storage periods [33]. All of these ingredients may have played a synergistic role in providing physical stability to the emulsion. It is speculated that the microalgae will be suspended in the intricate wall matrix of inulin, whey protein isolate and maltodextrin. The freeze-dried encapsulated samples were used as iron fortificants for the mice study, wheat flour shelf-life evaluation and sensory studies.

### 3.2. Growth Performance and Hematological Profile of Mice

**Figure 2:**
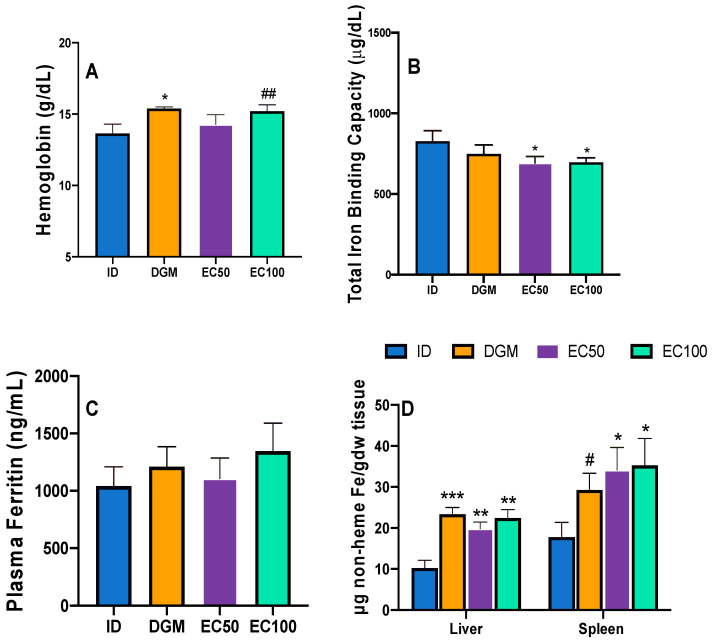
The effects of supplementing control diet (ID) with DGM or encapsulated DGM (EC50, EC100) on (**A**) hemoglobin; (**B**) total iron binding capacity; (**C**) plasma ferritin and (**D**) liver and spleen non-heme iron. Data are expressed as means ± S.E. Means with a * denote significant difference (*p* < 0.05) from the control; ** *p* < 0.01, *** *p* < 0.001, ^#^ *p* = 0.06, ^##^ *p* = 0.07, n ≥ 6.

There was no treatment-related difference in body weights or average daily feed intake (ADFI) between the mice fed any of the experimental diets (Table 3). This indicates that the diets with encapsulated DGM were well tolerated by the mice. Given that the animals had similar blood Hb concentrations at week 0, the consumption of DGM (*p* < 0.05, 15.39 ± 0.11 g/dL) and EC100 (*p* = 0.07, 15.21 ± 0.44 g/dL) diets enhanced blood Hb concentrations over the ID-fed mice (13.66 ± 0.64 g/dL) by week 4 (Figure 2A). However, the blood Hb concentrations of mice were not affected by the addition of EC50 to the diets. In response to iron depletion, TIBC is increased [34], under a physiological mechanism to upregulate iron sequestration for utilization in Hb synthesis. In this study, as compared to the control, the encapsulated DGM group (EC50 or EC100) had reduced (*p* < 0.05) TIBC values (Figure 2B). While the TIBC values in the DGM group was 16.1% lower than the control, this difference was not statistically significant (*p* = 0.12). Inclusion of DGM or encapsulated DGM in mouse diets resulted in moderate (DGM, 16.3%; EC50, 6.5%; EC100, 29.4%) but insignificant improvements in plasma ferritin levels (Figure 2C). Red pulp macrophages, present in the spleen, are primarily responsible for catabolizing senescent red blood cells (RBC). Erythrophagocytosis yields an estimated 20 μg Fe/day that is continuously recycled for new RBC production. However, under elevated hepcidin activity, non-heme iron in the spleen is increased (due to FPN degradation), sometimes to amounts greater than present in the liver [35,36,37,38,39,40]. Such an effect was also observed in the current investigation. Compared to ID, EC100 diet enhanced (*p* < 0.05) non-heme liver and spleen iron amounts by over two-fold (Figure 2D). Supplementation of EC50 to the control diet only added 9.6 mg Fe/kg, but its inclusion greatly improved (*p* < 0.05) non-heme liver and spleen iron contents over the control. While splenic non-heme iron contents in the DGM-fed mice were numerically higher than ID-fed mice, this difference was not significant (*p* = 0.06).

The enhanced bio-accessibility of iron through encapsulated DGM diets could be due to the disruption of microalgal cell walls [6,41,42], as a result of high-pressure homogenization. This suggests a relatively high bioavailability of iron through encapsulated DGM. Collectively, these results represent an improved iron status for the anemic mice consuming encapsulated DGM diets. Since EC100 had comparable hematological responses to DGM, this data demonstrates that encapsulation did not negatively affect the bioavailability of DGM iron and reinforces its potential as an iron fortificant in its current encapsulated form. This finding is novel, as to our best knowledge there is no literature available on the hematological effects of encapsulated DGM in animal models.

### 3.3. Effect of Storage on Ferrous Iron

The effects of storage of flours on ferrous iron are shown in Table 4. The addition of DGM-containing powders (EC/CM/DGM) enhanced the amount of total iron in the wheat flour. When compared to WF on day 0 at 20 °C, the total amount of ferrous iron in the flours was highest (*p* < 0.05) in CM100 samples (76.2 ± 0.1 μg) and was lowest (*p* < 0.05) in the positive control (DGM100, 64.6 ± 0.1 μg), in the 100% iron fortification category. Over time, storage of flours resulted in a decrease (*p* < 0.05) in ferrous iron in all treatments. At day 30 at 20 °C, the highest ferrous iron retention was found to be in DGM100 samples, with up to 1.5-fold higher (*p* < 0.05) values than WF.

Under accelerated degradation at 45 °C for 15 days, the amounts of ferrous iron reported in encapsulated sample EC50 was highest (*p* < 0.05) in its fortification category, showing a 96% higher amount than WF. At 30 days of storage, CM100 flour (53.2 ± 0.2 μg) observed the highest (*p* < 0.05) amounts of ferrous iron values. These levels were up to 1.2-fold higher (*p* < 0.05) than WF. Over the course of storage, WF observed a 36% drop (*p* < 0.05) in ferrous iron amounts. Such effects were likely to have been observed due to the oxidation of ferrous iron to its ferric form [43]. When wheat flour, fortified with ferrous sulfate and ethylenediamine tetraacetate, was stored at 30–35 °C for 42 days, it resulted in a significant decline in ferrous iron [15,44]. However, in this study, compared to WF, the encapsulated samples (EC50 or EC100) reduced oxidation and reported higher levels of ferrous iron content at the end of 30 days at both ambient and abusive temperatures. It has been shown that color-masking with TiO_2_ may provide an additional barrier to prevent iron oxidation [45], which was observed in our study for CM100 samples stored at both temperatures.

Given that the initial total iron content in the EC-fortified flour samples was lower than CM-fortified flours (50% fortification, up to 3.5 mg Fe/kg lower; 100% fortification, up to 10.2 mg Fe/kg lower), considerable benefit was observed with EC supplementation. On day 30 at 20 °C, EC50 samples were not different to CM- or DGM-fortified flours. However, for samples stored at 45 °C, while EC100 reported a higher value than DGM100, those values were only 6% lower than CM100. The ferrous iron oxidation from day 0 values was only 16% in EC100 samples, in comparison to up to 30% in CM/DGM-fortified flours. As our emulsion formulation also included citric acid, some of these effects may have been observed due to its chelating properties [46,47] as has been reported by others [48]. In conclusion, encapsulation provided modest protection against oxidation of ferrous iron to ferric iron. In terms of iron physiology, these results may hold relevance, as ferrous ions are more soluble than ferric ions and therefore, may have improved bioavailability [49].

### 3.4. Effect of Storage on Oxidative Stability

Since iron is a strong pro-oxidant, it promotes oxidation of the lipids present in the wheat flour, especially over longer storage periods [4]. MDA (malondialdehyde) is one of the secondary products formed as a result of lipid peroxidation and is a widely used biomarker of oxidative stress [50]. The MDA results were expressed as μmol/g crude fat to eliminate any confounding effects arising from different fat concentrations. After 15 days of storage at 20 °C, the average MDA values of EC50 and EC100 flours were 127.5 and 79.6 μmol/g crude fat, respectively (Table 5), 16.5% and 47.9% lower (*p* < 0.05) than WF at day 0. In contrast, WF saw a 15.5% increase (*p* < 0.05) in MDA amounts by day 15 over day 0. The highest (*p* < 0.05) MDA values were noted for DGM100 flour (47% increase over control at day 0). Further storage for 15 days (day 30) resulted in higher (*p* < 0.05) MDA levels across all treatments. When compared to WF at day 30, the lowest (*p* < 0.05) and highest (*p* < 0.05) μmol MDA/g crude fat values were obtained for EC100 and DGM100 flours (−44.9% and 24.5%, respectively). In the WF group, the mean MDA concentrations increased (*p* < 0.05) by 35.9% between day 0 and day 30.

A trend similar to that of the 20 °C samples was observed in samples stored at 45 °C (Table 5). On day 15, the encapsulated samples (EC50 or EC100) exhibited the lowest MDA amounts/g crude fat in their respective categories. Compared to day 0, DGM100 had the highest amount (263.6 ± 0.8 μmol/g fat) of MDA product formation, while EC100 sample had the lowest (75.9 ± 0.0 μmol/g fat) by day 30. In that time, WF saw an approximate 47.2% increase from baseline.

While, on a crude fat basis, encapsulated samples showed comparable or better results than other treatments at both ambient and abusive storage temperatures, on a μmol/g flour basis, encapsulated samples showed higher MDA amounts. Indeed, higher temperatures (>30 °C) and iron levels above 30 mg/kg can result in rancidity in flours [4]. However, this observation was not noted for EC50 samples stored at 45 °C, as it had less or comparable MDA amounts to other treatments. In this study, the higher oxidation values in encapsulated samples could be due to a few factors.

Firstly, there is evidence that lipid oxidation is directly correlated to fat content [51]. The fat content in the encapsulated samples was almost 1.9-fold to 3.5-fold higher than WF (Table S2). Secondly, the emulsions were formulated with soybean oil as the oil phase. Soybean oil contain 58.6% polyunsaturated fatty acids (PUFA), of which 53.1% is linoleic acid (C18:2) alone [52]. It has been estimated that the presence of two double bonds in a structure can promote oxidation by as much as 40 times over one double bond [53]. Thirdly, soy lecithin has a relatively high negative charge and thus attracts positively charged metal ions (Fe^2+^/Fe^3+^) to stimulate metal-induced oxidation of unsaturated fatty acids [54]. It has also been reported that the use of whey protein isolate to encapsulate iron can result in high peroxide values [55]. Lastly, ferrous ions are at least 100 times more oxidative than ferric ions [56]. That the encapsulated samples had a high amount of ferrous iron retention, together with the oxidation-prone PUFA profile of soybean oil [57] and the aforementioned causes, could have resulted in higher MDA amounts in the encapsulated samples. Compared to uncoated samples, ferrous fumarate, encapsulated in soy stearine, also caused higher fat oxidation when used to fortify ultra-rice [58]. Other studies have also shown that the addition of ferrous sources of iron to wheat flour resulted in oxidative degradation of linoleic acid and off-flavor development within 4–6 weeks of storage and was deemed unacceptable by a sensory panel [4]. We used unsaturated lipids to abide by the dietary guidelines and for their proven health benefits [59]; however, saturated fats may better mitigate adverse lipid peroxidation effects. In cases where PUFAs are added to commercial foods, suitable antioxidants, chelating agents, reduced oxygen packaging and (or) an appropriate wall matrix should also be included to prevent such effects [60].

### 3.5. Effect of Storage on Color Attributes of Fortified Wheat Flour

The effects of storage and temperature on L* (lightness) of WF and fortified flours are presented in Table 6. Upon addition of DGM-containing powders (DGM, EC, CM) to WF on day 0 at 20 °C, the lightness of the flours decreased (*p* < 0.05), as dark green-colored microalgae was added to the flour. While this effect was observed with all groups, EC50 (79.72 ± 0.12) was lighter and most similar in L values to WF (81.33 ± 0.10) than the other flours. Across treatments, the powders providing 17.5 mg of iron (50% RDA) were lighter than powders providing 35 mg of iron (100% RDA). By day 30, while EC50 had higher (*p* < 0.05) L* values than both CM and DGM, EC100 was darker (*p* < 0.5) compared to these groups.

Under accelerated storage at 45 °C, the encapsulated samples were the lightest in their respective fortification categories when compared to WF on day 15. However, by day 30, encapsulated samples had the lowest L* values. It is likely that such color changes were associated with increased MDA amounts as a result of accelerated fat oxidation [61]. In EC samples, heat application may have also triggered further interactions between the emulsified DGM and the food matrix. Another possible explanation could be due to Maillard browning of whey protein [62], especially stored under sealed pouches [63], resulting in off-color development. The color changes in whey protein can be expected due to the cross-interaction of the basic amino groups with sugars to form brown melanoidins [64]. The presence of polyphenols enhance color development by acting as a reducing agent in iron redox reactions [65]. However, in this case, that does not seem to be a likely event as color formation has not been consistent across DGM-containing treatments and does not seem to decline rapidly over time.

The effects of storage and temperature on a* (red-green) and b* coordinate (yellow-blue) of WF and fortified flours are presented in Table 7 and Table 8. When compared to WF at day 15, all samples but EC50 presented green color appearance (negative a* values). This trend was observed in samples stored at both 20 °C and 45 °C. Over the course of 30 days at 20 °C, EC50 performed better (*p* < 0.05) than the other treatment groups. However, these reported values were still different (*p* < 0.05) than WF. These data are consistent with high L* values for EC50 samples. Some of this color darkening in encapsulated products may be a result of a partial degradation of the capsule wall, thereby exposing the green color of DGM. Across groups fortified with 35 mg iron/kg (EC100/CM100/DGM100) at both temperatures, b* values were consistently higher (*p* < 0.05) than WF. For the same samples stored at either ambient or abusive temperature, EC100 had a* values closest to WF by the end of 30 days, again suggesting that encapsulation helped overcome some of the negative color attributes of DGM.

### 3.6. Effect of Storage on Moisture Content

The moisture content of the control and fortified flours were determined on 0, 15 and 30 days of storage. The results are presented in Supplementary Table S4. There was no significant difference in the moisture content at any time point between treatments during the storage period. The low moisture content in our samples and the barrier bags used prevented moisture migration and mold growth and may help in providing protection from insect damage, increasing storage stability of the fortified flours [66].

### 3.7. Sensory Study

**Figure 3:**
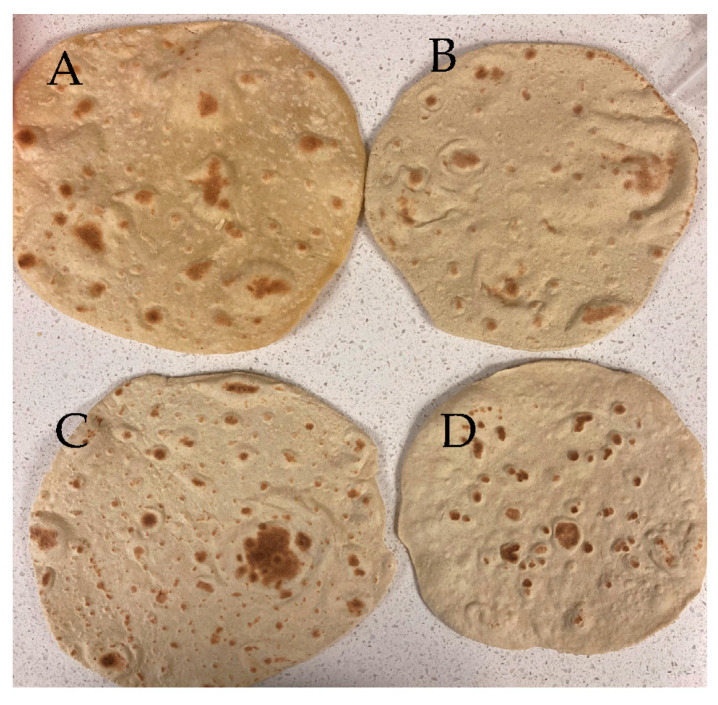
Roti (Indian flat bread) prepared from unfortified or fortified flour stored at room temperature for 21 days; (**A**) WF: whole wheat flour, (**B**) CM50: whole wheat flour + color-masked 17.5 mg iron roti, (**C**) EC50: whole wheat flour + encapsulated 17.5 mg iron roti and (**D**) EC100: whole wheat flour + encapsulated 35 mg iron.

**Figure 4:**
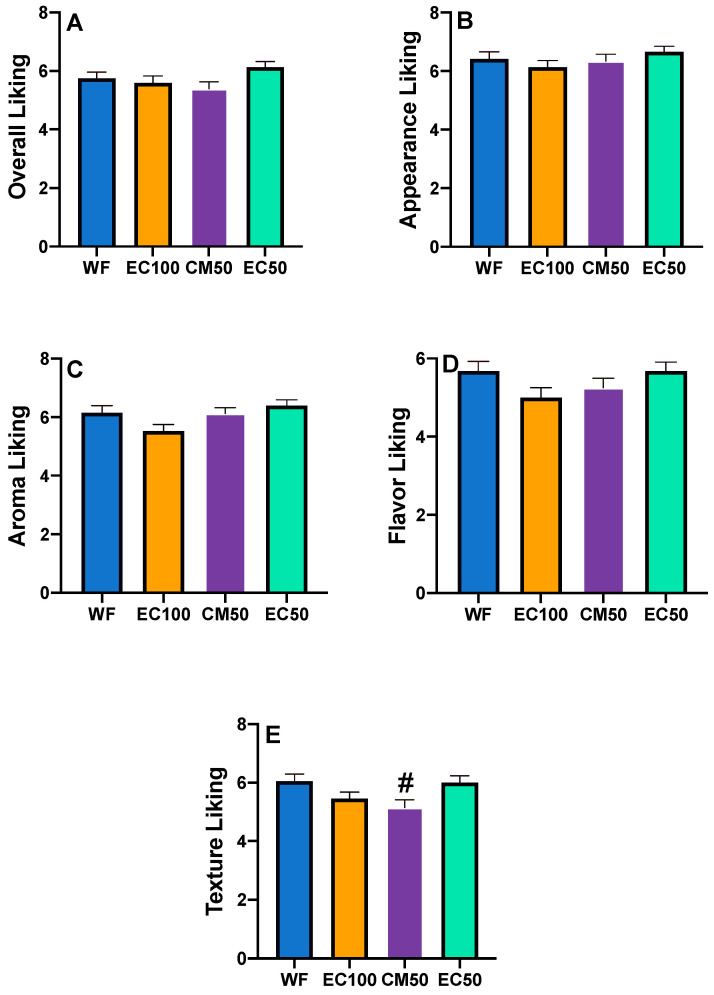
Hedonic scores (means ± S.E.) of whole wheat flour (WF) roti (Indian flat bread) samples fortified with encapsulated defatted *Nannochloropsis oceanica* (EC) and color-masked defatted *Nannochloropsis oceanica* (CM) day 0 on (**A**) Overall liking; (**B**) Appearance liking; (**C**) Aroma liking; (**D**) Flavor liking; and (**E**) Texture liking. ^#^ *p* = 0.06, n = 58.

**Figure 5:**
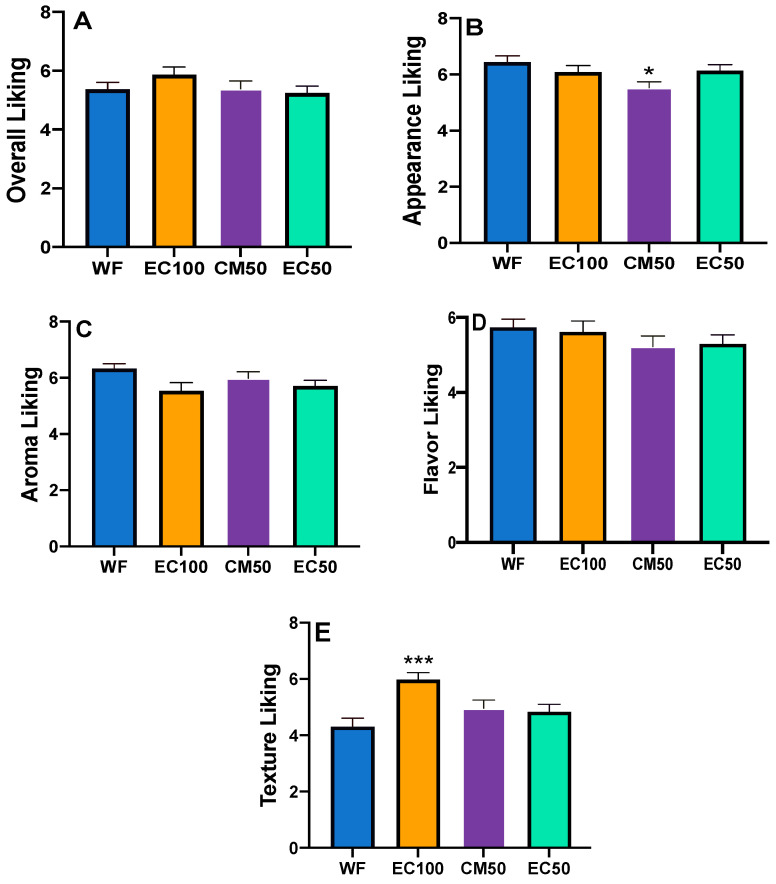
Hedonic scores (means ± S.E.) of whole wheat flour (WF) roti (Indian flat bread) samples fortified with encapsulated defatted *Nannochloropsis oceanica* (EC) and color-masked defatted *Nannochloropsis oceanica* (CM) at day 21 on (**A**) Overall liking; (**B**) Appearance liking; (**C**) Aroma liking; (**D**) Flavor liking; and (**E**) Texture liking. Means with a * denote significant difference (*p* < 0.05) than the control. *** *p* < 0.001, n = 51.

**Figure 6:**
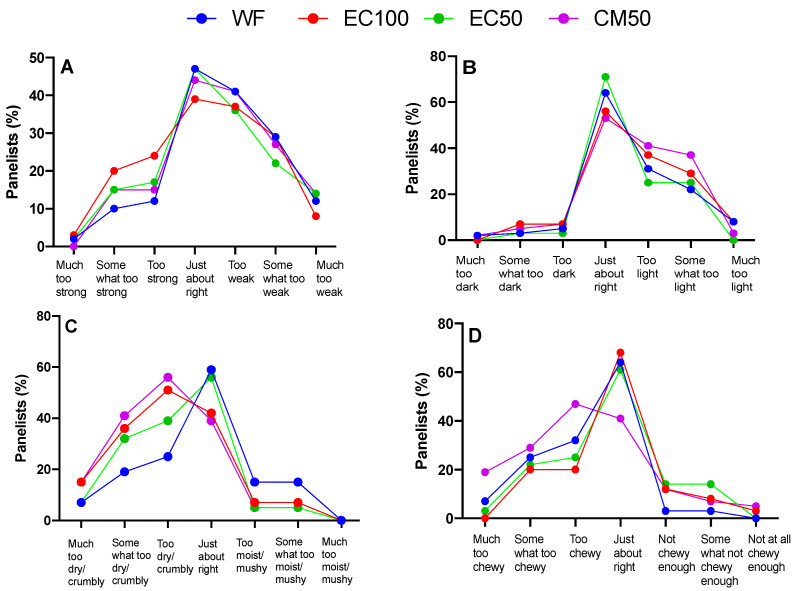
Just-about-right scales (% panelists) of whole wheat flour (WF) roti (Indian flat bread) samples fortified with encapsulated defatted *Nannochloropsis oceanica* (EC) and color-masked defatted *Nannochloropsis oceanica* (CM) at day 0 on (**A**) flavor, (**B**) color, (**C**) dry vs. moist and (**D**) chewiness; n = 58.

**Figure 7:**
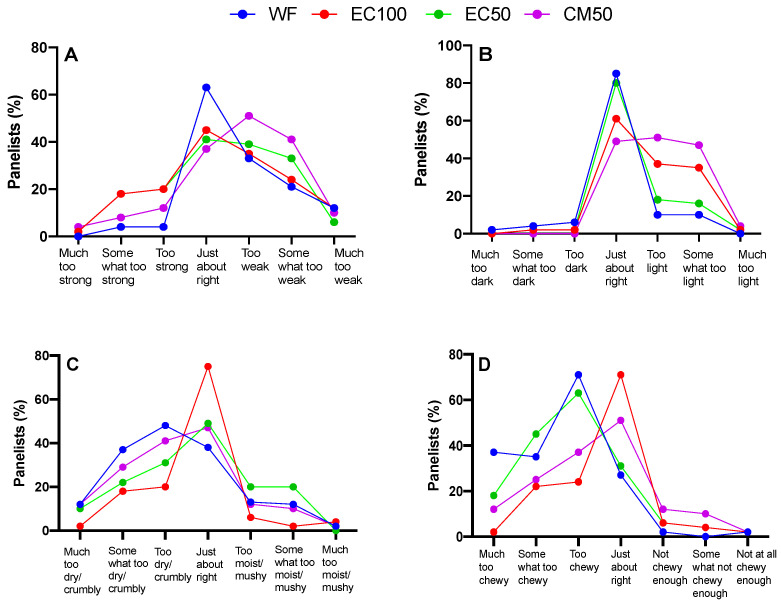
Just-about-right scales (% panelists) of whole wheat flour (WF) roti (Indian flat bread) samples fortified with encapsulated defatted *Nannochloropsis oceanica* (EC) and color-masked defatted *Nannochloropsis oceanica* (CM) at day 21 on (**A**) flavor, (**B**) color, (**C**) dry vs moist and (**D**) chewiness; n = 51.

**Figure 8:**
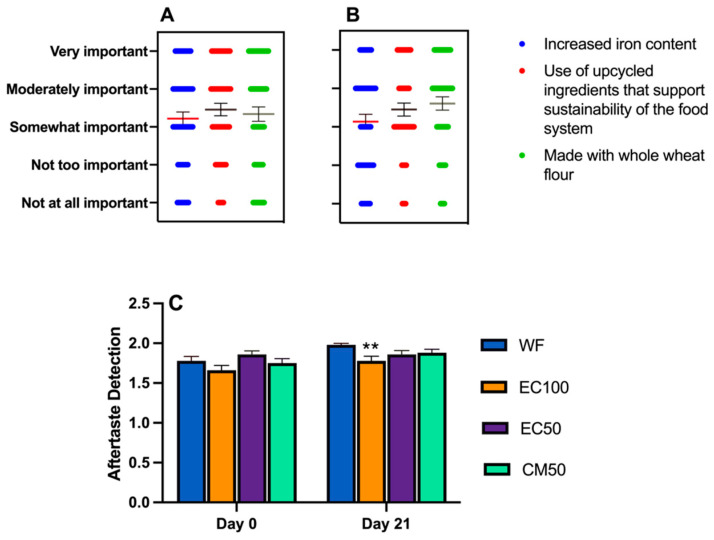
Claims provided for purchase intentions (means ± S.E.) during (**A**) day 0 and (**B**) day 21. A wider band corresponds to higher importance given by the panelist; (**C**) aftertaste detection (means ± S.E.) in whole wheat flour (WF) roti (Indian flat bread) samples fortified with encapsulated defatted *Nannochloropsis oceanica* (EC) and color-masked defatted *Nannochloropsis oceanica* (CM) on days 0 and 21; n = 58 (Day 0) and n = 51 (day 21); ** *p* < 0.01.

All hedonic attributes were characterized by normal distribution (Shapiro-Wilk test), while variance was homogeneous except for texture liking (Spearman’s test) (Supplementary Table S5). Based on a preliminary informal taste panel, DGM-fortified roti was clearly unacceptable and hence was not included in the sensory studies. Previous studies have shown that preparing rotis from wheat flour stored for longer than 60 days can adversely affect sensory attributes [67]. In the present work, there were no differences in the overall liking or appearance between rotis made from WF (control) or WF + EC at days 0 or 21 (Figure 3, Figure 4A,B and Figure 5A,B, respectively). In contrast, the appearance of rotis made from CM50 stored for 21 days were less liked in appearance than the control (Figure 5B). When the hedonic scores were analyzed for Indian panelists only (n = 15), there was no difference amongst treatments for any of the sensory attributes at day 0 (Figure S2, Supplementary). Color is strongly associated with quality of food [68]. In addition, there were no differences between WF and fortified rotis on flavor JAR or color JAR scales on day 0 (Figure 6A,B). While there were no JAR flavor differences between treatments by day 21, compared to the control, there was a significant (*p* < 0.05) decrease in JAR color for EC100 and CM50 stored flours (Figure 7B). In comparison to WF, there were no significant differences in fresh flour samples in aroma liking, chewiness or aftertaste detection on day 0 (Figure 4C, Figure 6D and Figure 8C respectively). For Indian panelists (n = 15) at day 0, the color JAR scores for EC50 were borderline higher (2.86 ± 0.13, *p* = 0.07) as compared to the control (2.40 ± 0.21) (Figure S3, Supplementary) and could be expected to become significant with a larger sample size. However, in flour samples stored at room temperature for 21 days, the aftertaste detection was higher (*p* < 0.05) in EC100 samples than WF (Figure 8C). In contrast, at day 21, EC100 and CM50 samples were less (*p* < 0.05) chewy than WF (Figure 7D). While there were significant color differences between flours using the L*a*b* color scheme upon fortificant addition at day 0, these changes were not visually detectable during the sensory trial and therefore did not significantly influence color JAR scores (Figure 6B). Meanwhile, by day 21, EC100 and CM50 samples had lower (*p* < 0.05) color JAR compared to WF (Figure 7B). The higher MDA values measured in encapsulated samples did not result in apparent off-flavor development in both fresh or stored flour samples (Figure 6A and Figure 7A, respectively). When compared to the control, EC50 reported comparable or better values on almost all tested sensory parameters in both fresh and stored flour samples. These data suggest that encapsulation was able to provide a physical barrier between DGM and the food matrix and resulted in an acceptable masking of the color, aroma, taste and flavor of the DGM. However, compared to WF (6.05 ± 0.03), CM50 reported lower (*p* = 0.06) texture liking values (5.15 ± 0.03) (Figure 4E) on day 0, conversely, EC100 reported higher (*p* < 0.05) texture liking values on day 21 (Figure 5E). Such higher (*p* < 0.05) texture values for EC100 samples were also obtained when results were analyzed for Indian panelists only (n = 9) (Figure S4, Supplementary). In addition, on day 21, the effect of addition of EC100 to whole wheat flour resulted in significant (all *p* < 0.05) improvements in responses from Indian panelists on flavor JAR, dry vs. moist JAR and chewiness JAR, in comparison to WF (Figure S5, Supplementary). There were no differences between the control or EC50 in texture at any time point. On day 0, while WF roti (3.17 ± 0.01) was perceived as equally moist to rotis prepared from EC50 samples (3.41 ± 0.01), the rotis prepared from CM50 (3.66 ± 0.01) and EC100 (3.59 ± 0.01) were perceived as significantly drier (*p* < 0.05) to WF rotis (Figure 6C). However, this effect was not observed with day 21 samples (Figure 7C). Rotis are most often applied with clarified butter (ghee) before serving or consumption, which enhances their appeal. Previous sensory studies on Indian bread (roti/chapati) have reported providing a side of vegetable curry [44,69]. In this study, the panelists were provided with rotis without any ghee or side-dish to counteract any additional flavors from those foods that may disguise the true flavors of the rotis. Rotis are typically consumed fresh, as they can become stale within a few hours [70] due to starch retrogradation, resulting in increased firmness and chewiness [71]. Due to the in-home evaluation process used, the panelists could report their responses up to 32 h after the preparation of this roti. As a result, the responses were received from 2 PM on the day of the experiments and the panelists continued to record responses until 8 PM the following day. Such long durations may have affected the quality of the roti. Another factor that may play a major role in determining the texture of the roti is the flour quality [72]. While the number of sensory participants in the study were modest, there is evidence that a base size of 40–60 panelists can result in reliable and robust averages and studies with 80 or more panelists do not significantly impact the averages on attributes (Moskowitz 1997) [73].

**Figure 9:**
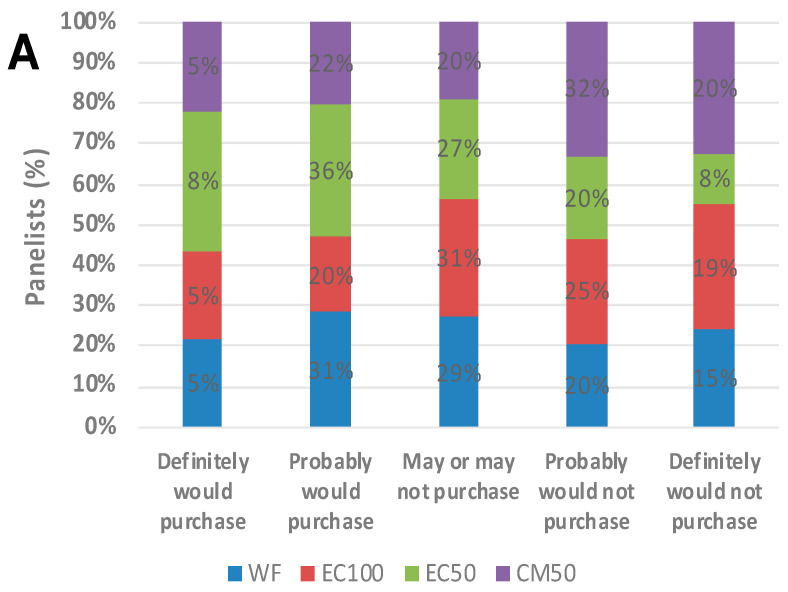

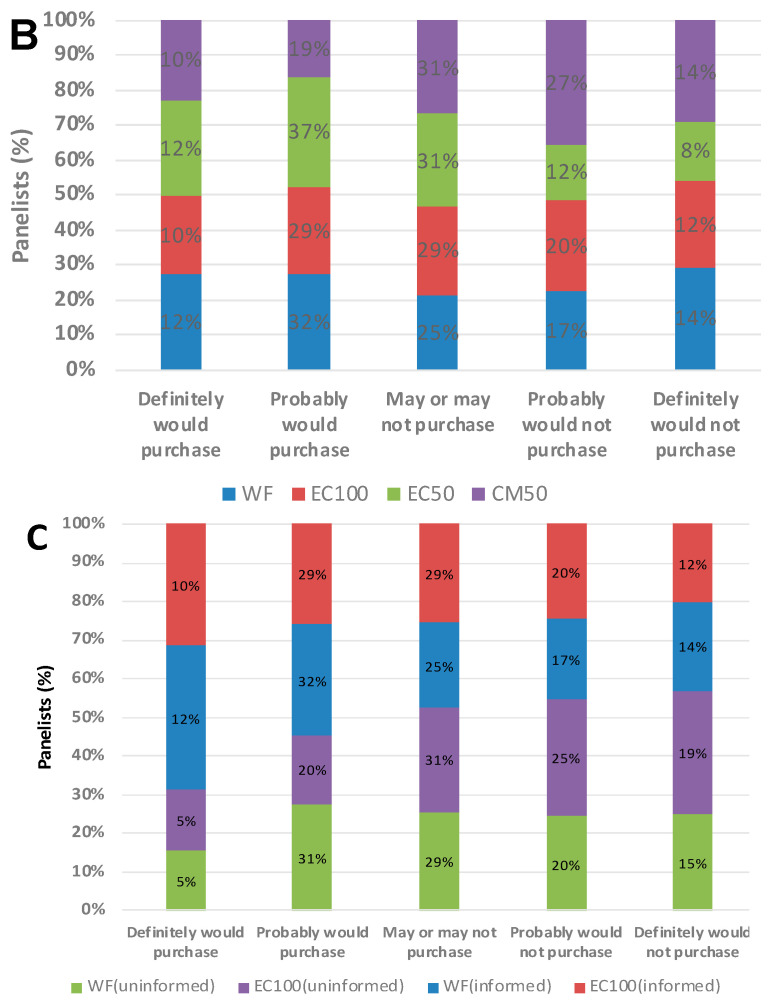
Purchase intent of whole wheat flour (WF) roti (Indian flat bread) samples fortified with encapsulated defatted *Nannochloropsis oceanica* (EC) and color-masked defatted *Nannochloropsis oceanica* (CM) at day 0, (**A**) pre-claim, (**B**) post-claim and (**C**) comparison between WF and EC100; n = 58.

**Figure 10:**
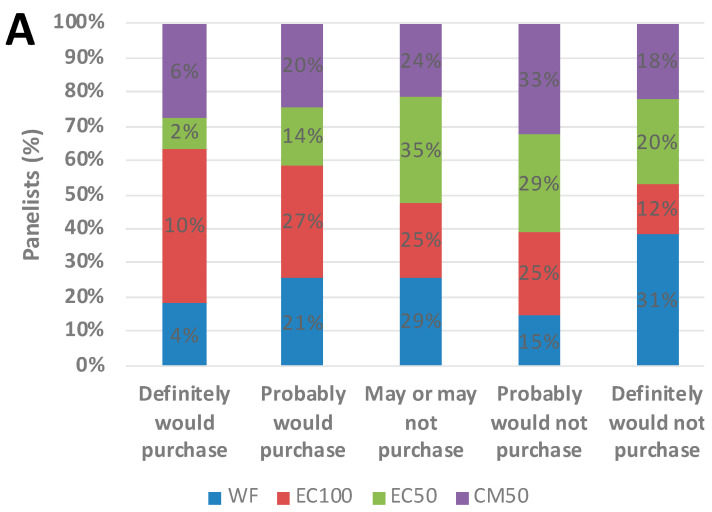

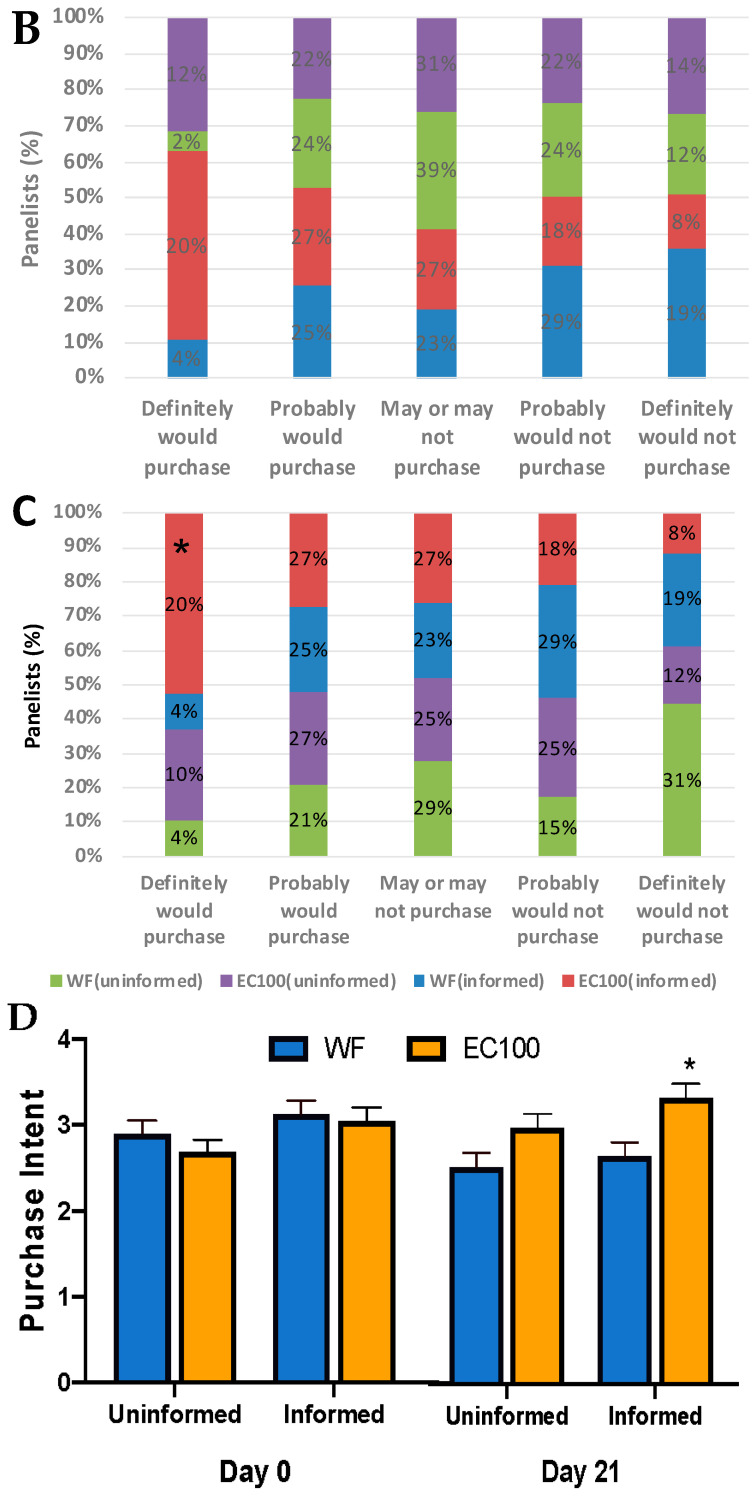
Purchase intent (means ± S.E.) of whole wheat flour (WF) roti (Indian flat bread) samples fortified with encapsulated defatted *Nannochloropsis oceanica* (EC) and color-masked defatted *Nannochloropsis oceanica* (CM) at day 21, (**A**) pre-claim, (**B**) post-claim, (**C**) comparison between WF and EC100 and (**D**) overall comparison between WF and EC100 on day 0 and day 21. * *p* < 0.05, n = 51.

Purchase intent information was also collected from the panelists before and after providing them with the information that the test samples were ‘prepared using upcycled ingredients that support sustainability of the food system’, ‘provided increased iron content’ and ‘made with whole wheat flour’. On day 0, compared to the control, the purchase intent of the consumers improved (*p* = 0.055) upon learning that the whole wheat roti encapsulated with 35 mg iron (EC100) provided 24% iron RDA for men and 11% iron RDA for women in a single serving of a 30 g roti (Figure 9B) and this intent was observed to be significant (*p* < 0.05) on day 21 for EC100 samples (Figure 10D). While there were only 59 panelists on day 0 study, potential significance could be expected with a larger sample size. For all other treatments, the purchase intent change remained insignificant.

**Figure 11:**
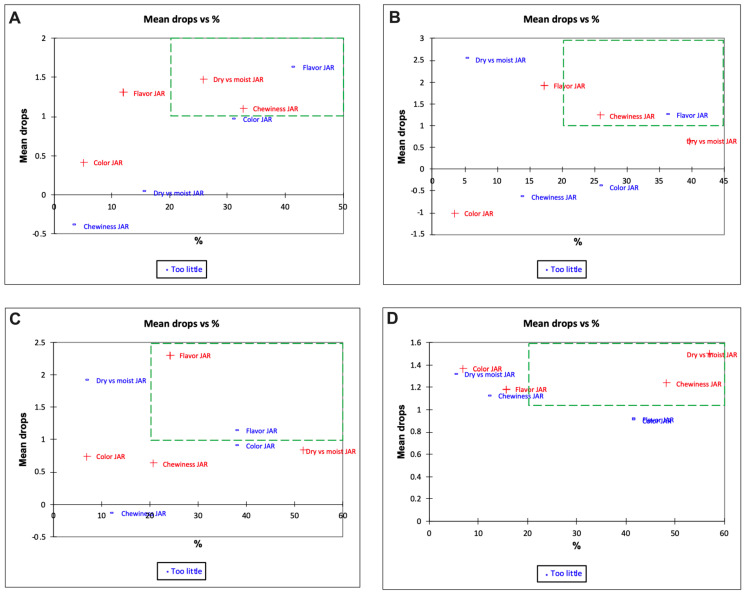
Penalty analysis of day 0 samples (mean drops vs. percent panelist) of Indian flat bread prepared from (**A**) control wheat flour (WF); (**B**) whole wheat flour + encapsulated 17.5 mg iron (EC50); (**C**) whole wheat flour + encapsulated 35 mg iron (EC100); and (**D**) whole wheat flour + color-masked 17.5 mg iron (CM50); n = 58.

**Figure 12:**
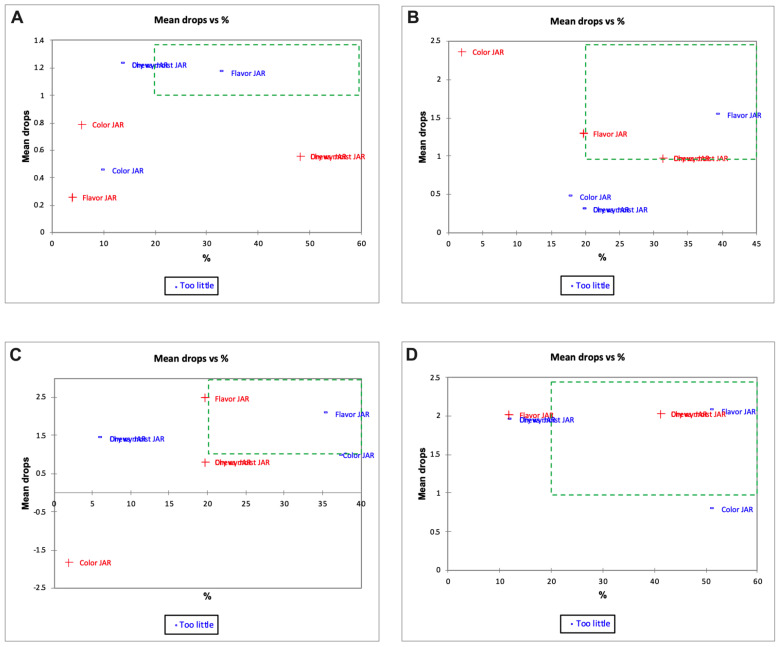
Penalty analysis of day 21 samples (mean drops vs. percent panelist) for Indian flat bread prepared from (**A**) control wheat flour (WF); (**B**) whole wheat flour + encapsulated 17.5 mg iron (EC50); (**C**) whole wheat flour + encapsulated 35 mg iron (EC100); and (**D**) whole wheat flour + color-masked 17.5 mg iron (CM50); n = 51.

A penalty analysis was also conducted to help identify attributes that most influenced consumer liking and also areas for improvement of these products [74]. In Figure 11 and Figure 12, the green zone can be considered as the critical zone requiring improvement. In the control sample, the panelists felt that the roti had too little flavor (Figure 11A and Figure 12A) and was dry (estimated 1.5 mean drop, Figure 11A). In normal practice, rotis are typically brushed with ghee and consumed with a side-dish, as mentioned previously [75]. Therefore, the panelists may not be accustomed to having the roti just by itself. Indeed, the dryness of the sample was not a concern for 15–20% of the panelists, suggesting that the diverse study population approached this product with different notions. The EC50 sample had more chewy bite (Figure 11B) and less flavor (Figure 11B and Figure 12B). Less flavor can mean that encapsulation overcame the objectionable flavor of the DGM. In addition, this sample also had improved color attributes (resulting in negative mean drops) (Figure 11B). Samples fortified with 35 mg iron (EC100) (Figure 11C and Figure 12C) had around 35–40% panelists who chose less flavor and 23% panelists who chose more flavor as critical attributes (Figure 11C). In this context, increased flavor is a bigger issue to address.

Future formulations can focus on improving the encapsulation formulation to allow for the addition of 35 mg iron while being able to successfully control the flavor. An optimized dough preparation process (kneading time, water addition, flour quality) may enhance the quality of the roti and ensure that it remains soft over a longer period. To meet the objectives of this study, the masking of color, taste, aroma and flavor of the DGM held primary importance. Our results demonstrate that the encapsulation method used was able to address those concerns.

## 4. Conclusions

This study provides first evidence that co-encapsulation of DGM with inulin using high pressure homogenization produced a stable emulsion, which after freeze-drying did not affect its iron bioavailability. We also exhibit that wheat flour, fortified with encapsulated DGM, showed an enhanced potential to prevent oxidation of ferrous iron. While EC-fortified flour stored for 30 days presented off-color development, those changes were considered acceptable, as verified through a sensory taste panel. The effect of any volatile compounds produced as a result of oxidative degradation did not affect product acceptance. The encapsulated products concealed the intense green color, fishy aroma and off-taste of DGM and resulted in products that were more shelf-stable than DGM alone. From these results, it appears that encapsulated DGM supplementation providing 17.5 mg iron, or 50% iron fortification rate, seem to exert maximum benefit when used to fortify wheat flour at both ambient and accelerated temperatures, without causing any sensory changes in cooked roti (Indian flat bread) prepared from both fresh and stored wheat flour samples. Our findings may offer new opportunities to promote the use of DGM as an iron fortificant in food applications (such as wheat flour) and can benefit millions of iron-deficient people in India who rely on monotonous staple diets for nourishment.

## Figures and Tables

**Table 1:** Amount of non-encapsulated (DGM), encapsulated (EC) and color-masked (CM) defatted *Nannochloropsis oceanica* added to wheat flour (WF, control) for shelf-life and sensory study *.

	Fortified 50% of Standard (17.5 mg Fe/kg) (% w/w)	Fortified 100% of Standard (35 mg Fe/kg) (% w/w)
Encapsulated DGM (EC)	4.375	8.750
Color-masked DGM (CM)	1.607	3.214
Defatted *Nannochloropsis oceanica* (DGM)	0.607	1.215

* For wheat flour, fortification standard at 100% fortification rate has been set between 28–42.5 mg iron for kg of flour [22].

**Table 2:** Particle size characteristics for o/w DGM-inulin emulsion stored at 4 °C.

Days	Volume Weighted Mean D(4,3) (μm)	Surface Weighted Mean D(3,2) (μm)
0	0.637 ± 0.002	0.119 ± 0.001
21	0.649 ± 0.007	0.119 ± 0.001
42	0.630 ± 0.004	0.119 ± 0.001

**Table 3:** Growth performance of mice fed different iron-fortified diets *.

		ID	DGM	EC50	EC100
Body weight (g)	Week 0	17.47 ± 0.64	17.98 ± 0.69	17.99 ± 0.59	17.71 ± 0.93
	Week 4	21.08 ± 0.92	20.63 ± 0.93	21.05 ± 0.97	20.12 ± 1.20
ADFI/mouse ^#^ (g)	Week 1	2.81 ± 0.16	2.65 ± 0.16	2.90 ± 0.14	2.54 ± 0.22
	Week 2	2.86 ± 0.07	2.80 ± 0.09	2.97 ± 0.12	2.68 ± 0.17
	Week 3	2.88 ± 0.13	2.65 ± 0.14	2.85 ± 0.15	2.53 ± 0.19
	Week 4	2.85 ± 0.18	2.75 ± 0.06	2.84 ± 0.16	2.78 ± 0.16
ADII/mouse ^##^ (μg)	Week 1	21.64 ± 1.27 ^a^	83.76 ± 5.05 ^b^	50.08 ± 2.43 ^c^	75.17 ± 6.49 ^b^
	Week 2	21.99 ± 0.54 ^a^	88.42 ± 2.82 ^b^	51.40 ± 2.15 ^c^	79.23 ± 5.17 ^b^
	Week 3	22.19 ± 1.03 ^a^	83.79 ± 4.49 ^b^	49.37 ± 2.62 ^c^	74.75 ± 5.62 ^b^
	Week 4	21.98 ± 1.39 ^a^	86.78 ± 1.74 ^b^	49.10 ± 2.85 ^c^	82.17 ± 4.83 ^b^

Data are expressed as means ± S.E. Means that do not share the same letter within the same row are significantly different (*p* < 0.05). * Iron-deficient (ID), ID + defatted green microalgae (DGM-fortified), ID + encapsulated DGM (50% fortification level or EC50 diet; 100% fortification level or EC100 diet). ^#^ Average Daily Feed Intake, ^##^ Average Daily Iron Intake.

**Table 4:** Effect of addition of non-encapsulated (DGM), encapsulated (EC) and color-masked (CM) defatted *Nannochloropsis oceanica* to wheat flour (WF, control) on ferrous iron (μg ferrous iron/g flour) ^1^.

Sample	Day 0	20 °C		45 °C	
		Day 15	Day 30	Day 15	Day 30
WF	37.2 ± 0.2	35.7 ± 0.2	21.1 ± 0.1	37.4 ± 1.6	23.9 ± 0.2
DGM50	55.1 ± 0.1 *	54.9 ± 0.8 *	35.7 ± 0.3 *	55.3 ± 0.2 *	42.7 ± 0.1 *
DGM100	64.6 ± 0.1 *	65.5 ± 0.4 *	54.4 ± 0.2 *	65.1 ± 0.1 *	47.3 ± 0.0 *
EC50	53.2 ± 0.1 *	63.5 ± 0.2 *	36.6 ± 0.3 *	73.2 ± 0.1 *	37.8 ± 0.6 *
EC100	66.0 ± 0.3 *	64.8 ± 0.3 *	44.9 ± 0.1 *	80.0 ± 0.5 *	50.2 ± 0.3 *
CM50	56.7 ± 0.6 *	55.9 ± 0.3 *	37.3 ± 0.1 *	54.4 ± 0.0 *	37.8 ± 0.2 *
CM100	76.2 ± 0.1 *	84.9 ± 0.2 *	53.1 ± 0.0 *	81.4 ± 0.3 *	53.2 ± 0.2 *

^1^ Data are expressed as means ± S.E. Within the same column, means denoted with a * are significantly different (*p* < 0.05) from the control; n = 2–3.

**Table 5:** Effect of addition of non-encapsulated (DGM), encapsulated (EC) and color-masked (CM) defatted *Nannochloropsis oceanica* to wheat flour (WF, control) on malondialdehyde levels (μmol MDA/g fat) ^1^.

Sample	Day 0	20 °C		45 °C	
		Day 15	Day 30	Day 15	Day 30
WF	152.8 ± 0.2	176.4 ± 1.2	209.1 ± 1.5	187.9 ± 0.2	225 ± 0.6
DGM50	139.5 ± 0.1 *	155.3 ± 0.9 *	203.3 ± 0.3	177.1 ± 0.3 *	211.0 ± 0.5 *
DGM100	145.8 ± 0.1 *	225.8 ± 1.1 *	259.1 ± 0.8 *	218.3 ± 2.6 *	263.6 ± 0.8 *
EC50	82.3 ± 0.1 *	127.5 ± 0.7 *	158.4 ± 1.2 *	115.4 ± 0.5 *	122.3 ± 0.7 *
EC100	43.4 ± 0.0 *	79.6 ± 2.3 *	93.3 ± 0.0 *	69.2 ± 0.1 *	75.9 ± 0.0 *
CM50	133.7 ± 0.1 *	162.1 ± 1.3 *	149.9 ± 0.3 *	164.9 ± 0.1 *	183.3 ± 3.5 *
CM100	160.4 ± 0.2 *	190.6 ± 2.6 *	186.3 ± 0.1 *	172.1 ± 0.1 *	218.4 ± 0.7 *

^1^ Data are expressed as means ± S.E. Within the same column, means denoted with a * are significantly different (*p* < 0.05) from the control; n = 2.

**Table 6:** Effect of addition of non-encapsulated (DGM), encapsulated (EC) and color-masked (CM) defatted *Nannochloropsis oceanica* to wheat flour (WF, control) on color coordinate L ^1^.

Sample	Day 0	20 °C		45 °C	
		Day 15	Day 30	Day 15	Day 30
WF	81.33 ± 0.10	80.75 ± 0.04	81.15 ± 0.00	80.17 ± 0.00	80.51 ± 0.14
DGM50	79.24 ± 0.27 *	79.06 ± 0.00 *	79.43 ± 0.01 *	78.74 ± 0.00 *	79.47 ± 0.01 *
DGM100	77.99 ± 0.10 *	77.96 ± 0.00 *	78.32 ± 0.00 *	77.35 ± 0.00 *	77.54 ± 0.00 *
EC50	79.72 ± 0.12 *	78.73 ± 0.01 *	79.58 ± 0.00 *	78.98 ± 0.01 *	78.06 ± 0.00 *
EC100	77.60 ± 0.01 *	77.37 ± 0.00 *	75.72 ± 0.01 *	77.93 ± 0.00 *	76.49 ± 0.01 *
CM50	78.55 ± 0.12 *	78.74 ± 0.01 *	78.95 ± 0.01 *	78.46 ± 0.00 *	78.77 ± 0.00 *
CM100	77.31 ± 0.04 *	77.24 ± 0.02 *	77.37 ± 0.01 *	77.33 ± 0.00 *	77.63 ± 0.00 *

^1^ Data are expressed as means ± S.D. Within the same column, means denoted with a * are significantly different (*p* < 0.05) from the control; n = 3.

**Table 7:** Effect of addition of non-encapsulated (DGM), encapsulated (EC) and color-masked (CM) defatted *Nannochloropsis oceanica* to wheat flour (WF, control) on color coordinate a ^1^.

Sample	Day 0	20 °C		45 °C	
		Day 15	Day 30	Day 15	Day 30
WF	1.20 ± 0.04	1.08 ± 0.02	1.16 ± 0.00	1.16 ± 0.01	1.15 ± 0.03
DGM50	−0.17 ± 0.01 *	−0.14 ± 0.01 *	−0.19 ± 0.01 *	−0.06 ± 0.00 *	−0.10 ± 0.01 *
DGM100	−0.79 ± 0.04 *	−0.72 ± 0.02 *	−0.70 ± 0.00 *	−0.49 ± 0.01 *	−0.38 ± 0.01 *
EC50	0.55 ± 0.05 *	0.43 ± 0.00 *	0.39 ± 0.01 *	0.34 ± 0.02 *	0.40 ± 0.01 *
EC100	0.08 ± 0.03 *	−0.05 ± 0.01 *	0.06 ± 0.01 *	−0.03 ± 0.01 *	−0.08 ± 0.01 *
CM50	0.04 ± 0.02 *	−0.31 ± 0.02 *	−0.26 ± 0.01 *	−0.17 ± 0.02 *	0.04 ± 0.02 *
CM100	−0.44 ± 0.02 *	−0.69 ± 0.02 *	−0.60 ± 0.01 *	−0.40 ± 0.01 *	−0.30 ± 0.00 *

^1^ Data are expressed as means ± S.D. Within the same column, means denoted with a * are significantly different (*p* < 0.05) from the control; n = 3.

**Table 8:** Effect of addition of non-encapsulated (DGM), encapsulated (EC) and color-masked (CM) defatted *Nannochloropsis oceanica* to wheat flour (WF, control) on color coordinate b ^1^.

Sample	Day 0	20 °C		45 °C	
		Day 15	Day 30	Day 15	Day 30
WF	11.46 ± 0.04	11.20 ± 0.04	11.67 ± 0.00	11.98 ± 0.00	12.26 ± 0.04
DGM50	12.61 ± 0.06 *	12.76 ± 0.00 *	12.90 ± 0.01 *	12.66 ± 0.00 *	12.83 ± 0.01 *
DGM100	13.53 ± 0.04 *	13.01 ± 0.01 *	13.40 ± 0.01 *	13.53 ± 0.01 *	13.82 ± 0.02 *
EC50	11.41 ± 0.13	11.87 ± 0.01 *	11.57 ± 0.01 *	12.80 ± 0.00 *	13.32 ± 0.01 *
EC100	12.54 ± 0.02 *	11.99 ± 0.01 *	12.40 ± 0.01 *	13.12 ± 0.01 *	13.86 ± 0.00 *
CM50	11.65 ± 0.03 *	11.97 ± 0.00 *	11.70 ± 0.00	12.11 ± 0.02 *	12.08 ± 0.00 *
CM100	11.16 ± 0.02 *	11.55 ± 0.01 *	11.76 ± 0.01	11.77 ± 0.01 *	11.95 ± 0.00 *

^1^ Data are expressed as means ± S.D. Within the same column, means denoted with a * are significantly different (*p* < 0.05) from the control; n = 3.

## Data Availability

The data presented in this study are available on request from the corresponding author.

## Supplementary Materials

The following supporting information can be downloaded at: https://www.mdpi.com/article/10.3390/foods12030675/s1, Table S1. Composition of experimental diets, Table S2. Moisture, dry matter, protein and fat content of experimental flours, Table S3. Panelist demographics, Table S4. Effect of encapsulation of moisture content of flour, Table S5. Normality and variance homogeneity test of variables, Figure S1. Experimental design of shelf-life study, Figure S2. Hedonic scores of Indian panelists on day 0, Figure S3. JAR scores (% Indian panelists) on day 0, Figure S4. Hedonic scores of Indian panelists on day 21, Figure S5. JAR scores (% Indian panelists) on day 21.

## References

[1] Kassebaum N.J., GBD 2013 Anemia Collaborators (2016). The Global Burden of Anemia. Hematol. Oncol. Clin. N. Am..

[2] Allen L., World Health Organization, Food and Agriculture Organization of the United Nations (2006). Guidelines on Food Fortification with Micronutrients.

[3] Mehansho H. (2006). Iron Fortification Technology Development: New Approaches. J. Nutr..

[4] Hurrell R.F. (1997). Preventing Iron Deficiency through Food Fortification. Nutr. Rev..

[5] Gouveia L., Batista A.P., Sousa I., Raymundo A., Bandarra N.M. (2008). Microalgae in Novel Food Products. Food Chemistry Research Developments.

[6] Becker E.W. (2007). Micro-Algae as a Source of Protein. Biotechnol. Adv..

[7] Palabiyik I., Durmaz Y., Öner B., Toker O.S., Coksari G., Konar N., Tamtürk F. (2018). Using Spray-Dried Microalgae as a Natural Coloring Agent in Chewing Gum: Effects on Color, Sensory, and Textural Properties. J. Appl. Phycol..

[8] Fradique M., Batista A.P., Nunes M.C., Gouveia L., Bandarra N.M., Raymundo A. (2013). Isochrysis Galbana and Diacronema Vlkianum Biomass Incorporation in Pasta Products as PUFA’s Source. LWT-Food Sci. Technol..

[9] Tańska M., Konopka I., Ruszkowska M. (2017). Sensory, Physico-Chemical and Water Sorption Properties of Corn Extrudates Enriched with Spirulina. Plant Foods Hum. Nutr..

[10] Manor M.L., Kim J., Derksen T.J., Schwartz R.L., Roneker C.A., Bhatnagar R.S., Lei X.G. (2017). Defatted Microalgae Serve as a Dual Dietary Source of Highly Bioavailable Iron and Protein in an Anemic Pig Model. Algal Res..

[11] Bhatnagar R.S., Miller D.D., Padilla-Zakour O.I., Lei X. (2020). Supplemental Microalgal Iron Helps Replete Blood Hemoglobin in Moderately Anemic Mice Fed a Rice-Based Diet. Nutrients.

[12] Marco E.R.D., Steffolani M.E., Martínez M., León A.E. (2018). The Use of *Nannochloropsis* sp. as a Source of Omega-3 Fatty Acids in Dry Pasta: Chemical, Technological and Sensory Evaluation. Int. J. Food Sci. Technol..

[13] Gheysen L., Lagae N., Devaere J., Goiris K., Goos P., Bernaerts T., Van Loey A., De Cooman L., Foubert I. (2019). Impact of *Nannochloropsis* sp. Dosage Form on the Oxidative Stability of n-3 LC-PUFA Enriched Tomato Purees. Food Chem..

[14] Bhatnagar R.S., Padilla-Zakour O.I. (2021). Plant-Based Dietary Practices and Socioeconomic Factors That Influence Anemia in India. Nutrients.

[15] Huma N., Rehman S.U., Awan J.A., Murtaza M.A., Arshad M.U. (2007). Effect of Packaging Materials on the Quality of Iron-Fortified Wholemeal Flour During Storage. J. Food Process. Preserv..

[16] Wegmüller R., Zimmermann M.B., Moretti D., Arnold M., Langhans W., Hurrell R.F. (2004). Particle Size Reduction and Encapsulation Affect the Bioavailability of Ferric Pyrophosphate in Rats. J. Nutr..

[17] Zimmermann M.B., Zuidam N.J., Nedovic V. (2010). Encapsulation of Iron and Other Micronutrients for Food Fortification. Encapsulation Technologies for Active Food Ingredients and Food Processing.

[18] Ravanfar R., Comunian T.A., Dando R., Abbaspourrad A. (2018). Optimization of Microcapsules Shell Structure to Preserve Labile Compounds: A Comparison between Microfluidics and Conventional Homogenization Method. Food Chem..

[19] Zhong Q., Jin M. (2009). Nanoscalar Structures of Spray-Dried Zein Microcapsules and in Vitro Release Kinetics of the Encapsulated Lysozyme as Affected by Formulations. J. Agric. Food Chem..

[20] National Research Council, Commission on Life Sciences, Food and Nutrition Board, Subcommittee on the Tenth Edition of the Recommended Dietary Allowance (1989). Recommended Dietary Allowances.

[21] South P.K., Lei X., Miller D.D. (2000). Meat Enhances Nonheme Iron Absorption in Pigs. Nutr. Res..

[22] Food Safety and Standards Authority of India (2017). Large-Scale Food Fortification in India: The Journey So Far and Road Ahead.

[23] Cassone D.R., Duffin M.A., Gannon D.L., Hansen T.S., Haynes L.C., Manns J.M., Pracek A., Worfolk P., Zhao B., Zhou N. (2012). Method to Extend Whole Grain Flour and Product Shelf Life.

[24] Ahmed M.S.H. (2015). Effect of Storage Temperature and Periods on Some Characteristics of Wheat Flour Quality. Food Nutr. Sci..

[25] Goyal P., Chugh L.K., Berwal M.K. (2017). Storage Effects on Flour Quality of Commonly Consumed Cereals. J. Appl. Nat. Sci..

[26] Tan S.Y., Yeung C.K., Tako E., Glahn R.P., Welch R.M., Lei X., Miller D.D. (2008). Iron Bioavailability to Piglets from Red and White Common Beans (*Phaseolus Vulgaris*). J. Agric. Food Chem..

[27] Kosse J.S., Yeung A.C., Gil A.I., Miller D.D. (2001). A Rapid Method for Iron Determination in Fortified Foods. Food Chem..

[28] Buege J.A., Aust S.D., Fleischer S., Packer L. (1978). [30] Microsomal Lipid Peroxidation. Methods in Enzymology.

[29] Hunter R.S. (1958). Photoelectric Color Difference Meter. J. Opt. Soc. Am..

[30] AOAC (2019). Official Methods of Analysis.

[31] Lawless H.T., Heymann H. (2010). Sensory Evaluation of Food: Principles and Practices.

[32] Ishii F., Sasaki I., Ogata H. (1990). Effect of Phospholipid Emulsifiers on Physicochemical Properties of Intravenous Fat Emulsions and/or Drug Carrier Emulsions. J. Pharm. Pharmacol..

[33] Akhtar M., Dickinson E. (2007). Whey Protein–Maltodextrin Conjugates as Emulsifying Agents: An Alternative to Gum Arabic. Food Hydrocoll..

[34] United Nations Children’s Fund, United Nations University, World Health Organization (2001). Iron Deficiency Anemia: Assessment, Prevention, and Control. A Guide for Programme Managers.

[35] Sonnweber T., Ress C., Nairz M., Theurl I., Schroll A., Murphy A.T., Wroblewski V., Witcher D.R., Moser P., Ebenbichler C.F. (2012). High-Fat Diet Causes Iron Deficiency via Hepcidin-Independent Reduction of Duodenal Iron Absorption. J. Nutr. Biochem..

[36] Aslam M.F., Frazer D.M., Faria N., Bruggraber S.F.A., Wilkins S.J., Mirciov C., Powell J.J., Anderson G.J., Pereira D.I.A. (2014). Ferroportin Mediates the Intestinal Absorption of Iron from a Nanoparticulate Ferritin Core Mimetic in Mice. FASEB J..

[37] Soe-Lin S., Apte S.S., Andriopoulos B., Andrews M.C., Schranzhofer M., Kahawita T., Garcia-Santos D., Ponka P. (2009). Nramp1 Promotes Efficient Macrophage Recycling of Iron Following Erythrophagocytosis in Vivo. Proc. Natl. Acad. Sci. USA.

[38] Yu Y., Jiang L., Wang H., Shen Z., Cheng Q., Zhang P., Wang J., Wu Q., Fang X., Duan L. (2020). Hepatic Transferrin Plays a Role in Systemic Iron Homeostasis and Liver Ferroptosis. Blood.

[39] Cherukuri S., Potla R., Sarkar J., Nurko S., Harris Z.L., Fox P.L. (2005). Unexpected Role of Ceruloplasmin in Intestinal Iron Absorption. Cell Metab..

[40] Marques O., Neves J., Horvat N.K., Colucci S., Guida C., Muckenthaler M.U. (2019). Iron-Related Parameters Are Altered Between C57BL/6N and C57BL/6J Mus Musculus Wild-Type Substrains. HemaSphere.

[41] Neumann U., Derwenskus F., Gille A., Louis S., Schmid-Staiger U., Briviba K., Bischoff S.C. (2018). Bioavailability and Safety of Nutrients from the Microalgae Chlorella Vulgaris, Nannochloropsis Oceanica and Phaeodactylum Tricornutum in C57BL/6 Mice. Nutrients.

[42] Zanella L., Vianello F. (2020). Microalgae of the Genus Nannochloropsis: Chemical Composition and Functional Implications for Human Nutrition. J. Funct. Foods.

[43] Martinez F.E., Vannucchi H. (1986). Bioavailability of Iron Added to the Diet by Cooking Food in an Iron Pot. Nutr. Res..

[44] Rehman S.U., Anjum S.A., Anjum F.M. (2006). Storage Stability of Ferrous Iron in Whole Wheat Flour Naan Production. J. Food Process. Preserv..

[45] Li Y.O., Diosady L.L., Wesley A.S. (2010). Iodine Stability in Iodized Salt Dual Fortified with Microencapsulated Ferrous Fumarate Made by an Extrusion-Based Encapsulation Process. J. Food Eng..

[46] Furia T.E., Chemical Rubber Company (1972). CRC Handbook of Food Additives.

[47] Ekholm P., Virkki L., Ylinen M., Johansson L. (2003). The Effect of Phytic Acid and Some Natural Chelating Agents on the Solubility of Mineral Elements in Oat Bran. Food Chem..

[48] Cho Y.-J., Alamed J., McClements D.J., Decker E.A. (2003). Ability of Chelators to Alter the Physical Location and Prooxidant Activity of Iron in Oil-in-Water Emulsions. J. Food Sci..

[49] McKie A.T., Barrow D., Latunde-Dada G.O., Rolfs A., Sager G., Mudaly E., Mudaly M., Richardson C., Barlow D., Bomford A. (2001). An Iron-Regulated Ferric Reductase Associated with the Absorption of Dietary Iron. Science.

[50] Ayala A., Muñoz M.F., Argüelles S. (2014). Lipid Peroxidation: Production, Metabolism, and Signaling Mechanisms of Malondialdehyde and 4-Hydroxy-2-Nonenal. Oxidative Med. Cell. Longev..

[51] Estévez M., Ventanas S., Cava R. (2005). Physicochemical Properties and Oxidative Stability of Liver Pâté as Affected by Fat Content. Food Chem..

[52] Kozłowska M., Gruczyńska E. (2018). Comparison of the Oxidative Stability of Soybean and Sunflower Oils Enriched with Herbal Plant Extracts. Chem. Pap..

[53] Szterk A., Roszko M., Sosińska E., Derewiaka D., Lewicki P.P. (2010). Chemical Composition and Oxidative Stability of Selected Plant Oils. J. Am. Oil Chem. Soc..

[54] Wang G., Wang T. (2008). Oxidative Stability of Egg and Soy Lecithin as Affected by Transition Metal Ions and PH in Emulsion. J. Agric. Food Chem..

[55] Cengiz A., Kahyaoglu T., Schröen K., Berton-Carabin C. (2019). Oxidative Stability of Emulsions Fortified with Iron: The Role of Liposomal Phospholipids. J. Sci. Food Agric..

[56] Choe E., Min D.B. (2006). Mechanisms and Factors for Edible Oil Oxidation. Compr. Rev. Food Sci. Food Saf..

[57] de Alencar E.R., Faroni L.R.D., Peternelli L.A., da Silva M.T.C., Costa A.R. (2010). Influence of Soybean Storage Conditions on Crude Oil Quality. Rev. Bras. Eng. Agríc. Ambient..

[58] Li Y., Diosady L.L., Jankowski S. (2008). Stability of Vitamin B1 in Ultra Rice® in the Presence of Encapsulated Ferrous Fumarate. Int. J. Food Sci. Nutr..

[59] Lunn J., Theobald H.E. (2006). The Health Effects of Dietary Unsaturated Fatty Acids. Nutr. Bull..

[60] Mcclements D.J., Decker E.A. (2000). Lipid Oxidation in Oil-in-Water Emulsions: Impact of Molecular Environment on Chemical Reactions in Heterogeneous Food Systems. J. Food Sci..

[61] Lund M.N., Ray C.A. (2017). Control of Maillard Reactions in Foods: Strategies and Chemical Mechanisms. J. Agric. Food Chem..

[62] Davies C.G.A., Netto F.M., Glassenap N., Gallaher C.M., Labuza T.P., Gallaher D.D. (1998). Indication of the Maillard Reaction during Storage of Protein Isolates. J. Agric. Food Chem..

[63] Kim M.N., Saltmarch M., Labuza T.P. (1981). Non-Enzymatic Browning of Hygroscopic Whey Powders in Open Versus Sealed Pouches. J. Food Process. Preserv..

[64] Saltmarch M., Labuza T. (1982). Nonenzymatic Browning via the Maillard Reaction in Foods. Diabetes.

[65] Mellican R.I., Li J., Mehansho H., Nielsen S.S. (2003). The Role of Iron and the Factors Affecting Off-Color Development of Polyphenols. J. Agric. Food Chem..

[66] Delcour J., Hoseney R.C. (2010). Storage of Cereals. Principles of Cereal Science and Technology.

[67] Akhtar S., Anjum F.M., Anjum M.A. (2011). Micronutrient Fortification of Wheat Flour: Recent Development and Strategies. Food Res. Int..

[68] Clydesdale F.M. (1997). Color: Origin, Stability, Measurement, and Quality. Food Storage Stability.

[69] Alam S., Shah H.U., Saleemullah, Riaz A. (2007). Comparative Studies on Storage Stability of Ferrous Iron in Whole Wheat Flour and Flat Bread (Naan). Int. J. Food Sci. Nutr..

[70] Parimala K.R., Sudha M.L. (2015). Wheat-Based Traditional Flat Breads of India. Crit. Rev. Food Sci. Nutr..

[71] Sidhu J.S., Al-Saqer J., Al-Zenki S. (1997). Comparison of Methods for the Assessment of the Extent of Staling in Bread. Food Chem..

[72] Zanoni B., Peri C., Pierucci S. (1993). A Study of the Bread-Baking Process. I: A Phenomenological Model. J. Food Eng..

[73] Moskowitz H. (1997). Base Size in Product Testing: A Psychophysical Viewpoint and Analysis. Food Qual. Prefer..

[74] Plaehn D., Horne J. (2008). A Regression-Based Approach for Testing Significance of “Just-about-Right” Variable Penalties. Food Qual. Prefer..

[75] Hegde S., Nair L.P., Chandran H., Irshad H. (2018). Traditional Indian Way of Eating—An Overview. J. Ethn. Foods.

