# Supplementary material for: Iron from Co-Encapsulation of Defatted Nannochloropsis Oceanica with Inulin Is Highly Bioavailable and Does Not Impact Wheat Flour Shelf Life or Sensorial Attributes
Source: Foods. 2023 Feb 3;12(3):675. doi: 10.3390/foods12030675 (PMC9914652; doi:10.3390/foods12030675)
Supplement: Supplementary file 1 [file foods-12-00675-s001.zip › foods-2148255-supplementary.pdf]

Table S1. Composition of the experimental diets (in % w/w).

Diet	Iron-Deficient (ID)	DGM-Fortified (DGM)	Fortified 50% by encapsulated DGM (EC50)	Fortified 100% by encapsulated DGM (EC100)
DGM [a]	–	1.14	–	–
Encap. DGM [b]	–	–	3.75	8.25
Rice	37.50	37.50	37.50	37.50
Casein	16.40	15.84	15.80	15.15
Corn starch	24.59	24.04	23.19	21.43
Corn oil	4.98	4.94	3.24	1.14
Mineral mix [c]	3.50	3.50	3.50	3.50
Vitamin mix [d]	1.00	1.00	1.00	1.00
Maltodextrin	10.00	10.00	10.00	10.00
Salt	0.83	0.83	0.83	0.83
Calcium phosphate	0.24	0.25	0.25	0.27
Calcium carbonate	0.08	0.07	0.07	0.06
Potassium citrate	0.33	0.33	0.33	0.33
Methionine	0.24	0.23	0.23	0.22
Choline bitartrate	0.20	0.20	0.20	0.20
Magnesium oxide	0.08	0.08	0.08	0.08
Calculated values				
ME, kcal/g	3.70	3.70	3.60	3.60
Crude protein, %	17.00	17.00	17.00	17.10
Crude fat, %	5.20	5.20	5.10	5.20
Fe, mg/kg	7.70	31.60	17.30	29.60

[a] Defatted *Nannochloropsis oceanica* (Fe: 2,700 mg/kg)
[b] Encapsulated DGM (Fe: 400 mg/kg)
[c] Envigo 06053
[d] AIN-93-VX Envigo 94047

Table S2. Moisture, dry matter, protein and fat content of experimental flours (% w/w).

	% Moisture	% Dry Matter	% Crude Protein	% Fat
Whole wheat flour	9.6	90.4	14.6	2.1
Whole DGM*, 50% fortification	9.4	90.6	14.9	2.4
Whole DGM, 100% fortification	9.4	90.6	15.0	2.0
Encapsulated DGM, 50% fortification	9.3	90.7	14.6	3.9
Encapsulated DGM, 100% fortification	9.0	91.0	14.5	7.4
Color-masked DGM, 50% fortification	9.6	90.4	14.8	2.3
Color-masked DGM, 100% fortification	9.3	90.7	14.9	2.2

*DGM, defatted *Nannochloropsis oceanica*

Table S3. Collected panelist demographics for the sensory evaluation of Indian flat bread.

		Day 0	Day 21
Total number of panelists		59	53
Gender	Male (%)	33.9	36.0
	Female (%)	62.7	60.0
	Non-conforming (%)	1.7	0.0
	Prefer not to say (%)	1.7	4.0
Mean age (years)		34.6	32.6
Age range (years)		21 - 71	19 - 71

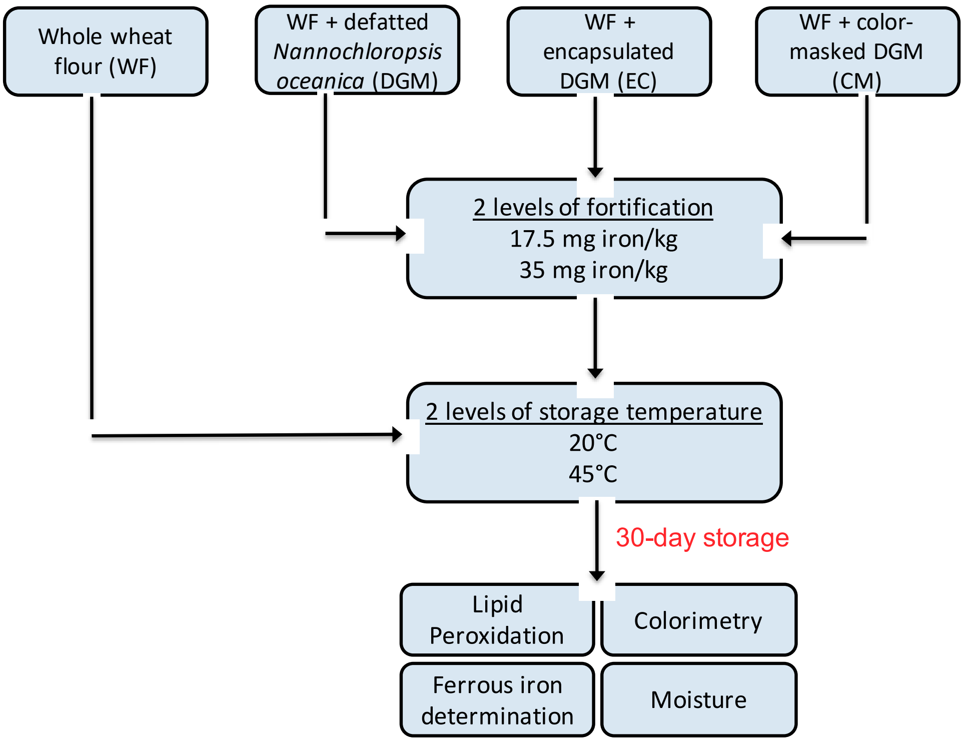

Figure S1. Experimental design for the shelf-life study of whole wheat flour (WF) fortified with defatted *Nannochloropsis oceanica* (DGM); encapsulated DGM (EC); or color-masked DGM (CM), at 50% and 100% iron fortification rates, and stored at 20°C or 45°C for 30 days.

Table S4. Effect of addition of non-encapsulated (DGM), encapsulated (EC) and color-masked (CM) defatted *Nannochloropsis oceanica* to wheat flour (WF, control) on moisture content (%w/w)[1].

Sample	Day 0	20°C		45°C	
		Day 15	Day 30	Day 15	Day 30
WF	6.12±0.14	6.72±0.35	5.64±0.09	5.47±0.12	5.29±0.13
DGM50	5.67±0.15	5.58±0.04	5.91±0.24	5.82±0.21	4.81±0.20
DGM100	5.84±0.03	5.63±0.81	4.21±1.44	5.56±0.13	4.51±0.10
EC50	5.20±0.78	5.47±0.32	5.36±0.23	5.82±0.11	5.23±0.00
EC100	5.10±0.31	5.70±0.02	5.10±0.00	4.17±0.93	4.22±0.08
CM50	5.38±0.48	5.99±0.06	4.85±0.58	5.84±0.23	4.62±0.85
CM100	5.38±0.10	5.77±0.19	5.17±0.27	5.51±0.28	4.63±0.02

[1]Data are expressed as means ± S.E. Within the same column, means denoted with a * are significantly different (P<0.05) from the control; n = 2.

Table S5. Normality (Shapiro-Wilk) and Variance Homogeneity (Spearman's rho) test of variables

Variable	P-value	rho value
Appearance	0.3625	-0.1255
Aroma	0.6246	-0.1394
Flavor	0.7459	-0.1532
Texture	0.9889	-0.0314*
Overall Liking	0.0939	-0.2157

*heterogeneous variance (p = 0.3275)

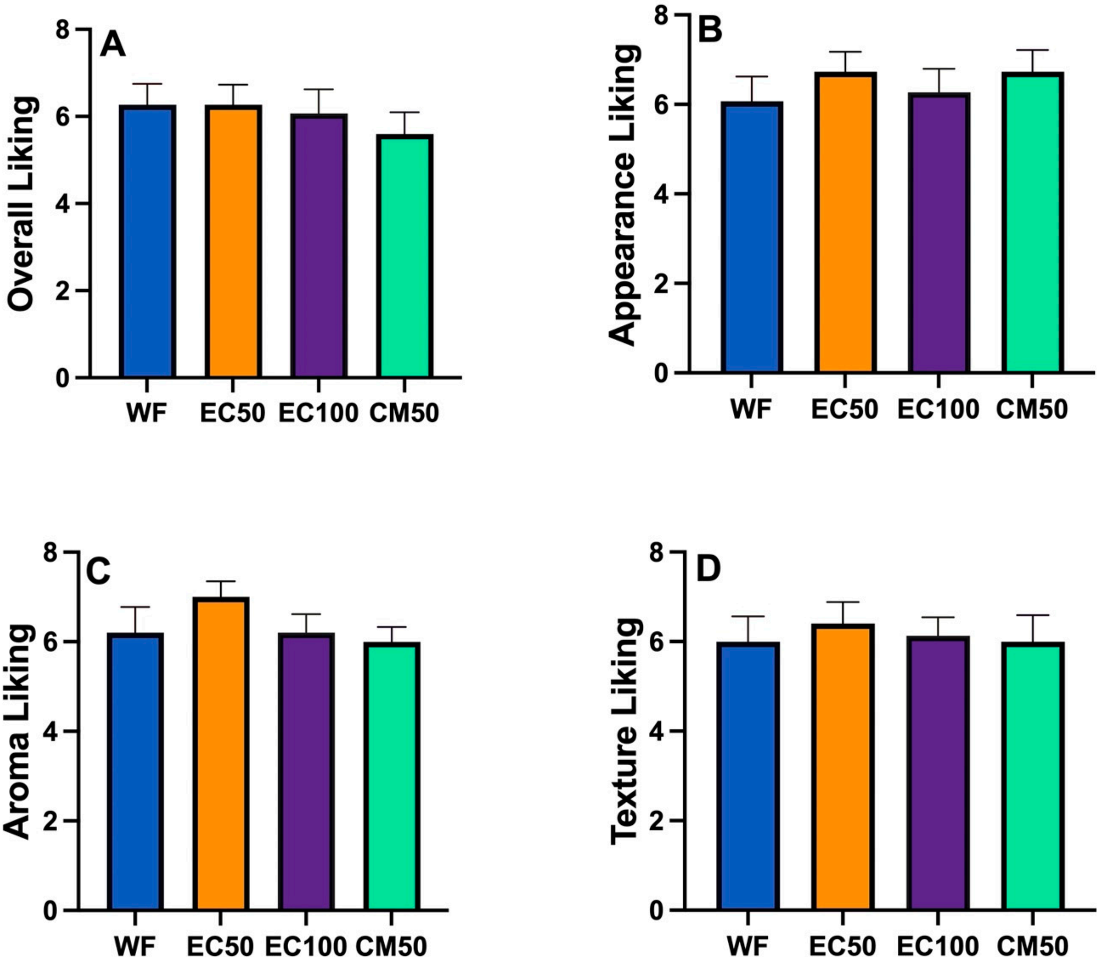

Figure S2. Hedonic scores of Indian panelists consuming whole wheat flour (WF) samples fortified with encapsulated defatted *Nannochloropsis oceanica* (EC) and color-masked defatted *Nannochloropsis oceanica* (CM) at day 0 on **(A)** Overall liking; **(B)** Appearance liking; **(C)** Aroma liking and **(D)** Texture liking. Means with a * denote significant difference (P<0.05) than the control. ***P<0.001, n = 15.

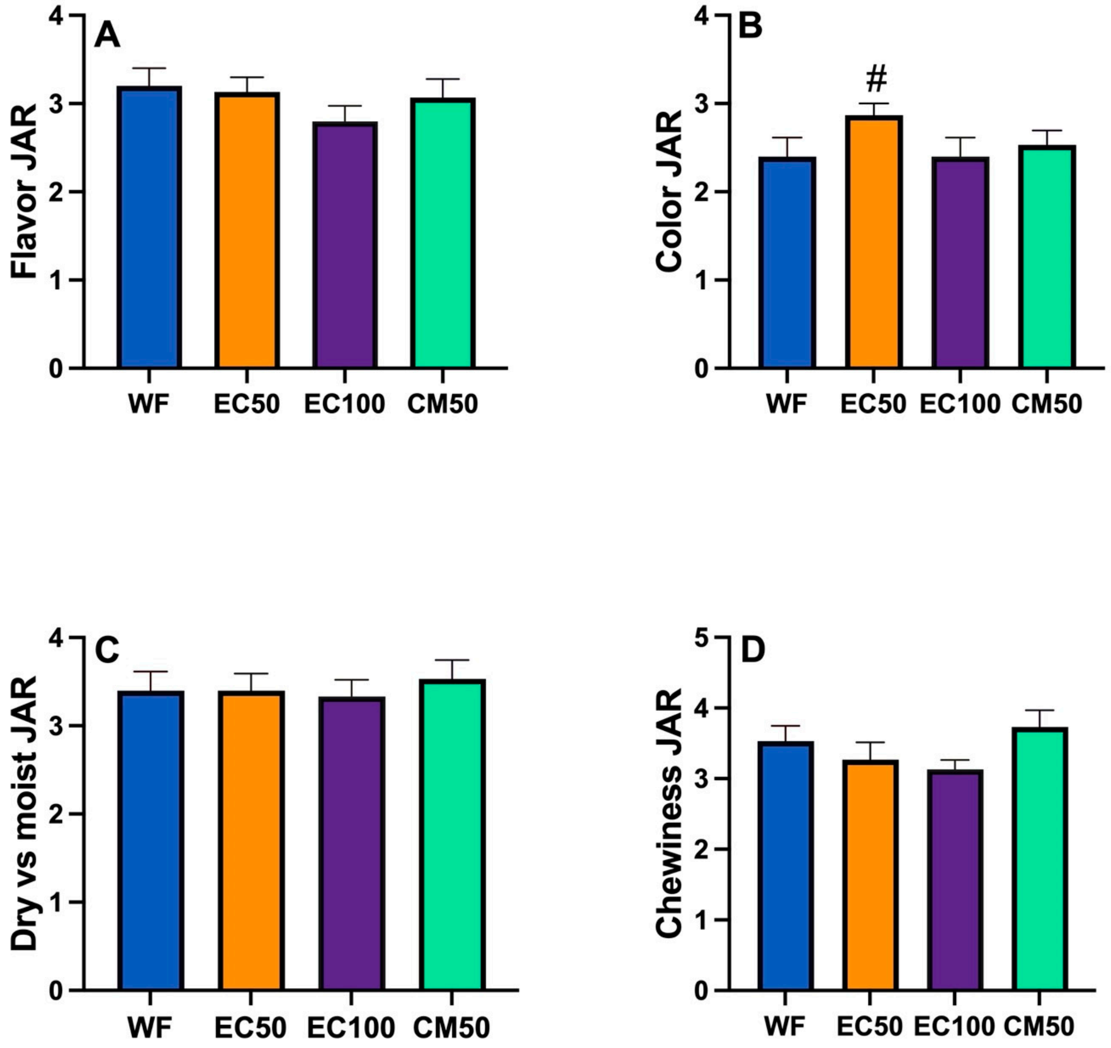

Figure S3. Just-about-right scales (% Indian panelists) of whole wheat flour (WF) samples fortified with encapsulated defatted *Nannochloropsis oceanica* (EC) and color-masked defatted *Nannochloropsis oceanica* (CM) at day 0 on **(A)** flavor, **(B)** color, **(C)** dry vs moist, and **(D)** chewiness. P = 0.07, n = 15.

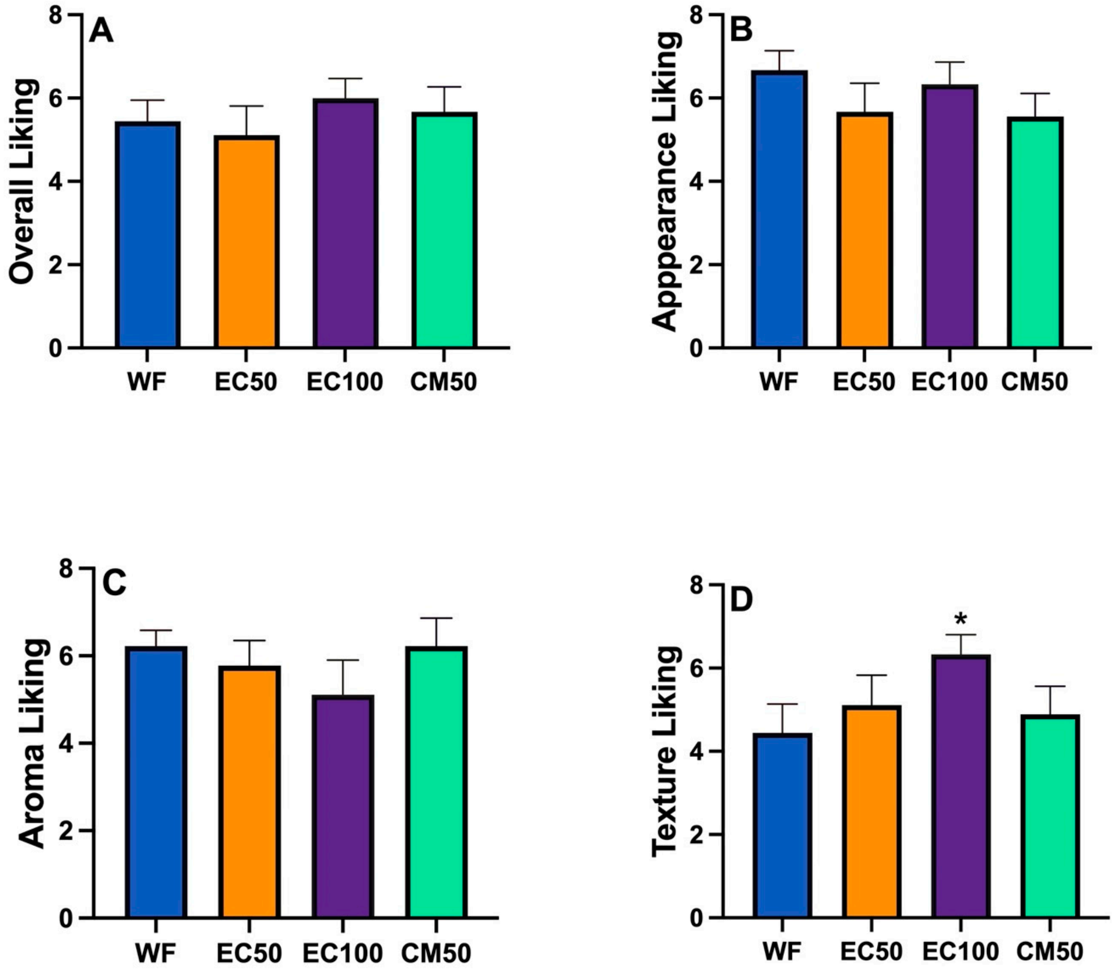

Figure S4. Hedonic scores of Indian panelists consuming whole wheat flour (WF) samples fortified with encapsulated defatted *Nannochloropsis oceanica* (EC) and color-masked defatted *Nannochloropsis oceanica* (CM) at day 21 on **(A)** Overall liking; **(B)** Appearance liking; **(C)** Aroma liking and **(D)** Texture liking. Means with a * denote significant difference (P<0.05) than the control. n = 9.

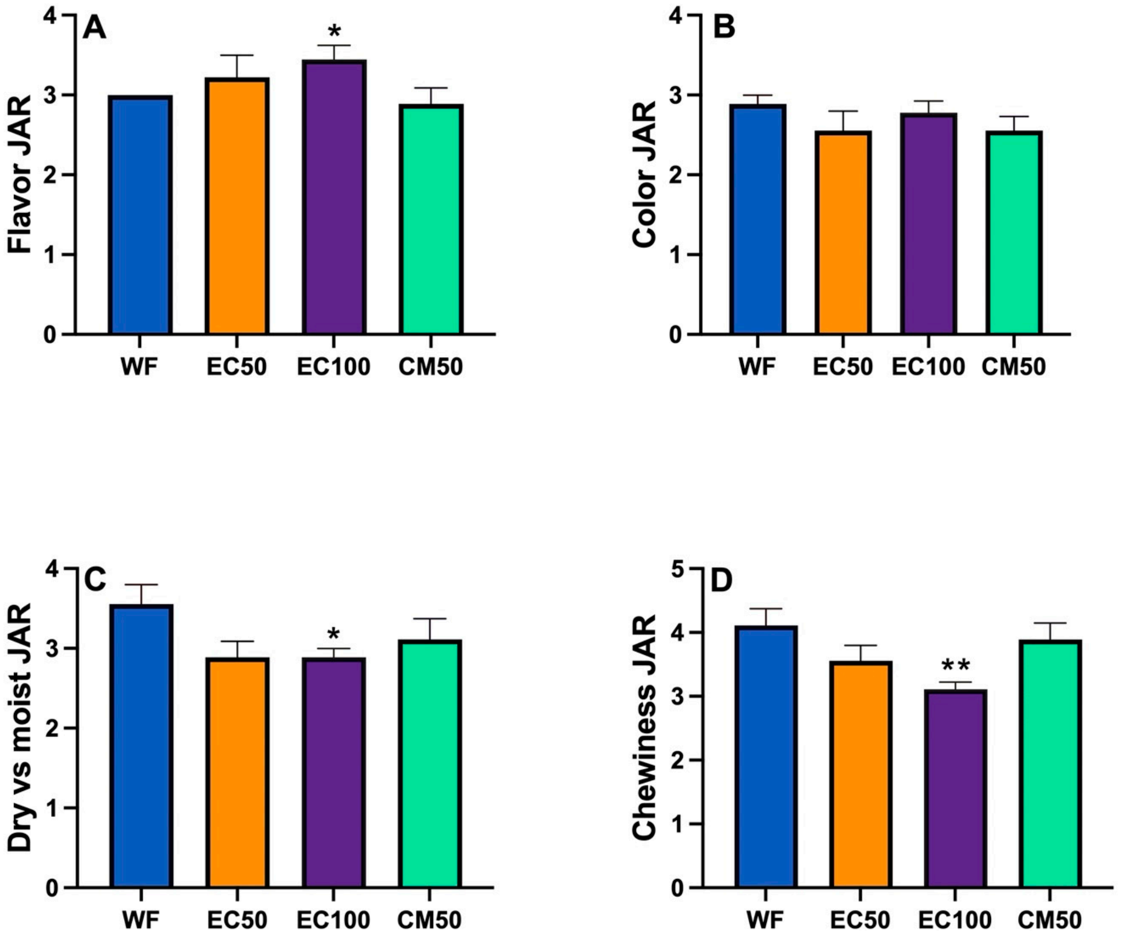

Figure S5. Just-about-right scales (% Indian panelists) of whole wheat flour (WF) samples fortified with encapsulated defatted *Nannochloropsis oceanica* (EC) and color-masked defatted *Nannochloropsis oceanica* (CM) at day 21 on **(A)** flavor, **(B)** color, **(C)** dry vs moist, and **(D)** chewiness. Means with a * denote significant difference ($P<0.05$) than the control. $^{**}P<0.01$, n = 9.
